# The human medial temporal lobe represents memory items in their ordinal position in both declarative and motor memory domains

**DOI:** 10.1371/journal.pbio.3003267

**Published:** 2025-07-07

**Authors:** Ainsley Temudo, Nina Dolfen, Bradley R. King, Genevieve Albouy

**Affiliations:** 1 Department of Health and Kinesiology, College of Health, University of Utah, Salt Lake City, Utah, United States of America; 2 Department of Psychology, Columbia University, New York, New York, United States of America; 3 Department of Experimental Psychology, Ghent University, Ghent, Belgium; International School for Advanced Studies, ITALY

## Abstract

Memory systems in humans are less segregated than initially thought as learning tasks from different memory domains (e.g., declarative versus procedural) can recruit similar brain areas. However, it remains unclear whether the functional role of these overlapping brain regions – and the hippocampus in particular – is domain-general. Here, we test the hypothesis that the hippocampus encodes and preserves the temporal order of sequential information irrespective of the nature of that information. We used multivariate pattern analyses (MVPA) of functional magnetic resonance imaging (fMRI) data acquired during the execution of learned sequences of movements and objects to assess brain patterns related to procedural and declarative memory processes, respectively. We also tested whether the hippocampus represents information about temporal order of items (here movements and objects in the motor and declarative domains, respectively) in a learned sequence irrespective of their nature. We also examined such coding in brain regions involved in both motor (primary and premotor cortices) and object (perirhinal cortex and parahippocampus) sequence learning. Our results suggest that hippocampal and perirhinal multivoxel activation patterns do not carry information about specific items or temporal position in a random series of objects or movements. Rather, these regions code for the representation of items in their learned temporal position in sequences irrespective of their nature (i.e., item-position coding). In contrast, although all other ROIs showed evidence of item-position coding, this representation could – at least partially – be attributed to the coding of other information such as position information. Altogether, our findings indicate that regions in the medial temporal lobe represent the temporal order of sequential information similarly in both the declarative and the motor memory domains. Our data suggest that these regions contribute to the development of item-position maps that might provide a cognitive framework for sequential behaviors irrespective of their nature.

## Abstract 2

Different memory domains can recruit similar brain areas, but it is not known if the functional role of these overlapping regions is shared or domain-specific. This neuroimaging study shows that the function of the hippocampus to preserve the order of sequential behaviors is shared across memory domains.

## Introduction

Influential models of memory organization suggest that the declarative and procedural memory systems are supported by distinct brain networks in humans [1]. This framework was largely based on neuropsychological studies showing that patients with hippocampal lesions exhibited severe declarative learning deficits but could learn new procedural (motor) skills [2,3]. However, more recent evidence suggests that these two memory systems are less segregated than initially thought [4,5]. For example, a large number of neuroimaging studies indicate that the hippocampus is involved in motor sequence learning^.^ (e.g., [6–12]) and that these hippocampal responses are related to the subsequent memory consolidation process [7,9]. This is in line with more recent studies in patients with hippocampal lesions demonstrating that the hippocampus is necessary for consolidation even when it is not required for initial learning [13,14]. Despite the evidence reviewed above suggesting that different memory systems can recruit common brain areas, it remains unclear whether the functional role of these overlapping brain regions – in particular the hippocampus – is shared across memory domains.

Indirect evidence for potential overlapping hippocampal function between memory domains comes from studies using multivariate analyses of functional Magnetic Resonance Imaging (fMRI) data. This research suggests that the hippocampus plays a critical role in the processing of temporal information in both the declarative (e.g., [15]) and the procedural memory domain [16]. In the declarative domain, results show that hippocampal activity patterns contain information about the temporal order of episodic events [17] as well as items in learned sequences of letters [18] and objects [15]. Recent work from our group has extended these findings to the procedural memory domain and has shown that hippocampal activation patterns carry information about the position of finger movements in a learned motor sequence [16]. These findings are in line with a recent influential model of hippocampal function, i.e., the *hippocampal sequencing hypothesis*, which proposes that the hippocampus produces a “*sequential content-free structure*” to organize experiences distributed across different cortical nodes that might represent *content-specific* representations [19]. This theory conceptualizes the hippocampus as “*a general-purpose sequence generator that carries content-limited ordinal structure, and tiles the gaps between events or places to be linked*” [20]. Based on this theory and the evidence reviewed above, it is tempting to speculate that the capacity of the hippocampus to encode the order of learned information does not depend on the nature of the learned items to be ordered. This, however, remains hypothetical as there is no empirical evidence supporting such view.

The aim of this study was therefore to empirically test this hypothesis. To do so, we designed a serial reaction time task (SRTT) that allowed us to probe sequence learning in both the motor and the declarative memory domains (i.e., memorizing a sequence of finger movements and a sequence of objects, respectively). We used multivoxel representational similarity analysis (RSA) of fMRI data acquired during sequence task performance to examine whether hippocampal activation patterns carry information about the order of items in a learned sequence irrespective of the memory domain. Based on the evidence presented above, we hypothesized that hippocampal activation patterns would represent information about the temporal order of learned items in a sequence within each memory domain (i.e., separately for movements [16] and objects [15]), but also regardless of the memory domain (i.e., irrespective of whether the item in a particular position is a movement or an object). We also examined such coding in brain regions involved in motor (primary motor and premotor cortices, [16]) and object (perirhinal cortex and parahippocampus, [15]) sequence learning. We expected item (finger/object) and position information (temporal position in the task stream) to be coded in memory domain-specific regions. Specifically, we hypothesized that fingers and objects would be coded in M1 and in the perirhinal cortex, respectively and that position information would be coded in the premotor and parahippocampal cortices [15,16].

## Results

**Fig 1:**
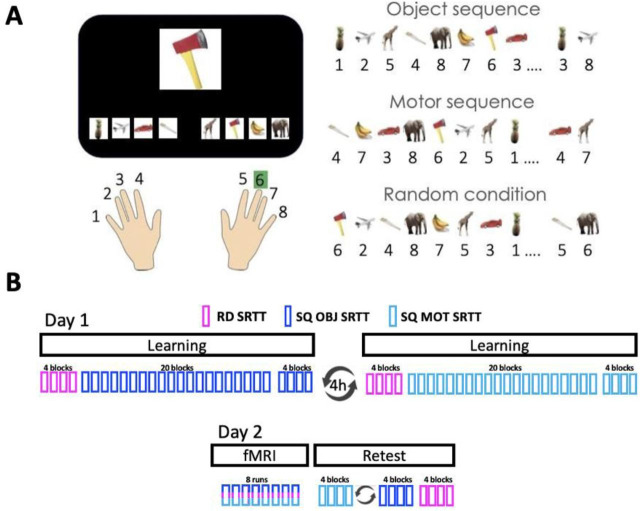
Serial reaction time task (SRTT) and Experimental Design. **(A)** Left panel. A stimulus appears in the center of the screen and participants are instructed to respond as fast and as accurately as possible according to the finger/object mapping displayed at the bottom of the screen. Note that the finger-object mapping displayed on the bottom of the screen changed after each stream of 8 elements (i.e., every 8 objects/8 finger presses) in order to orthogonalize object and motor sequence learning (see right panel). Right panel. Three different task conditions were designed: object sequence with random finger presses (that resulted in the learning of an 8-element object sequence, SQ OBJ), motor sequence with random object presentation (that resulted in the learning of an 8-element finger sequence, SQ MOT) and a random series with random finger presses and random object presentation (RD). Numbers in the figure represent fingers whereby 1 and 8 correspond to the left and right little fingers, respectively. All object images were derived from an open access online database at http://olivalab.mit.edu/MM/uniqueObjects.html [21]. The picture of the hands is taken from an open access online sharing service at https://www.clker.com. **(B)** Top panel. On day 1, after baseline performance assessment with a random (RD) SRTT, participants learned a sequence of objects (SQ OBJ SRTT) and a sequence of movements (SQ MOT SRTT), outside the MRI scanner, in two separate sessions 4h apart (order counterbalanced across participants). Bottom panel. On day 2, brain activity was recorded on the three task conditions with fMRI over 8 runs during which the RD, SQ OBJ and SQ MOT conditions were pseudo-randomly interleaved so that the same condition did not repeat more than 2 times in a row. A retest session was then performed outside the scanner to assess memory retention on each sequence condition (order counterbalanced across participants) followed by a final test on the random condition.

Participants performed a serial reaction time task (SRTT, Fig 1A) and learned, on the first experimental day, a sequence of finger movements (motor sequence, SQ MOT) and a sequence of objects (object sequence, SQ OBJ) during two separate experimental sessions the order of which was counterbalanced across individuals (Fig 1B). On day 2 of the experiment, participants’ brain activity was recorded using fMRI while they practiced the learned motor and object sequences. At the start of each experimental session on day 1, participants practiced a random control task (RD SRTT) involving random movements and random object presentation to measure baseline performance. This task was also performed in the scanner on day 2 to provide a control condition for the RSA (Fig 1A–1B). Performance on the three task conditions was retested outside the scanner on day 2 to probe memory retention (Retest, Fig 1B).

### Behavioral results

**Fig 2:**
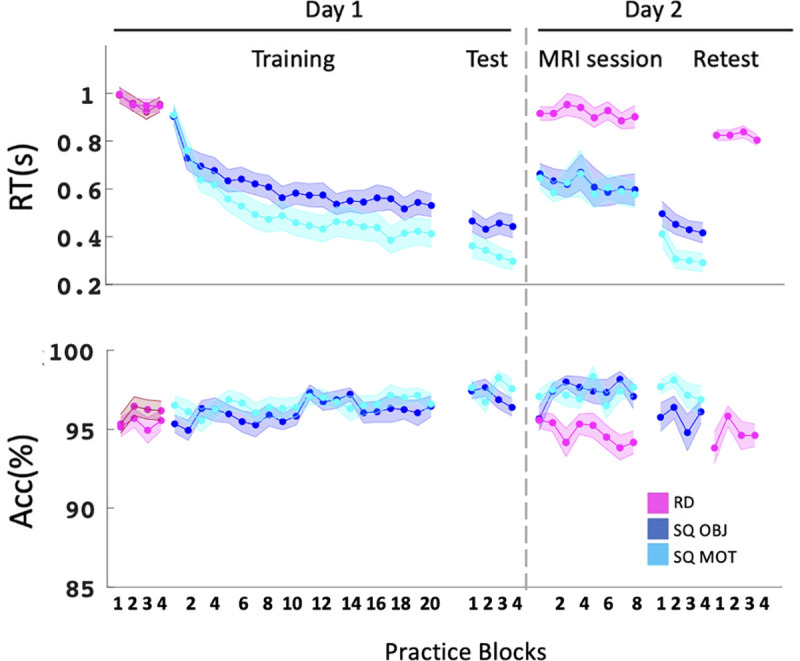
Behavioral results. Response time in seconds (top panel) and accuracy (% correct response, bottom panel) across blocks and sessions for all task conditions. Dark blue: object sequence (SQ OBJ), light blue: motor sequence (SQ MOT), pink: random blocks (RD) (darker shade corresponds to random practice during the object session). Shaded error bars = SEM. The source data corresponding to the top and bottom panels are included in S1 Data, “Fig 2” sheet.

Analyses of baseline performance extracted from the random SRTT acquired at the start of each learning session (Fig 1) are reported in Section A in S1 Text. Analyses of the sequence SRTT data indicated that performance speed (i.e., mean response time) improved during learning on day 1 for both the motor and object sequence tasks (block effect; training: *F*(19,551) = 38.98, *ɳ*_*p*_^2^ = 0.57, *p* < 0.001; test: *F*(3,87) = 3.78, *ɳ*_*p*_^2^ = 0.12, *p* = 0.01) but the motor task presented overall faster performance (condition by block effect; training: *F*(19,551) = 2.72, *ɳ*_*p*_^2^ = 0.09, *p* < 0.001; test: *F*(3,87) = 1.57, *η*_*p*_^2^ = 0.05, *p* = 0.2; condition effect; training: *F*(1,29) = 5.65, *η*_*p*_^2^ = 0.16, *p* = 0.02; test: *F*(1,29) = 9.45, *ɳ*_*p*_^2^ = 0.25, *p* = 0.005; Fig 2, day 1, top panel). In contrast, performance accuracy remained stable during learning on day 1 and similar between conditions (training: condition by block effect: *F*(19,551) = 0.61, *ɳ*_*p*_^2^ = 0.02, *p* = 0.9; block effect: *F*(19,551) = 1.43, *ɳ*_*p*_^2^ = 0.05, *p* = 0.11; condition effect: *F*(1,29) = 3.25, *ɳ*_*p*_^2^ = 0.1, *p* = 0.08; test: condition by block effect: *F*(3,87) = 2.84, *ɳ*_*p*_^2^ = 0.09, *p* = 0.04; block effect *F*(3,87) = 0.72, *ɳ*_*p*_^2^ = 0.02, *p* = 0.55; condition effect: *F*(1,29) = 1.2, *ɳ*_*p*_^2^ = 0.04, *p* = 0.28; Fig 2, day 1, bottom panel).

Data collected on day 2 in the MRI scanner indicated that performance remained stable across runs (block effect; speed: *F*(7,203) = 0.92, *ɳ*_*p*_^2^ = 0.03, *p* = 0.5; accuracy: block effect: *F*(7,203) = 1.23, *ɳ*_*p*_^2^ = 0.04, *p* = 0.29). Importantly, performance was better for the sequence conditions as compared to random condition (condition effect; speed: *F*(2,58) = 66.39, *ɳ*_*p*_^2^ = 0.7, *p* < 0.001; motor versus random: *p* < 0.001; object versus random: *p* < 0.001; accuracy: *F*(2,58) = 26.28, *ɳ*_*p*_^2^ = 0.48, *p* < 0.001; motor versus random: *p* < 0.001; object versus random: *p* < 0.001) but similar between sequence conditions (speed and accuracy: object versus motor: *ps* > 0.99; Fig 2, MRI session). During the retest performed outside the scanner, performance speed improved across practice blocks (block effect: *F*(3,87) = 8.52, *ɳ*_*p*_^2^ = 0.23, *p* < 0.001) while accuracy remained stable (block effect: *F*(3,87) = 2.8, *ɳ*_*p*_^2^ = 0.09, *p* = 0.05). Importantly, performance was overall better for sequence conditions when compared to random (condition effect; speed: *F*(2,58) = 126.8; *ɳ*_*p*_^2^ = 0.81, *p* < 0.001; motor versus random: *p* < 0.001; object versus random: *p* < 0.001; accuracy: condition effect; *F*(2,58) = 8.31, *ɳ*_*p*_^2^ = 0.22, *p* < 0.001; motor versus random: *p* < 0.002; object versus random: *p* = 0.31) and performance for the motor task remained better than for the object task (motor versus object; speed: *p* = 0.001; accuracy: *p* = 0.08; Fig 2, Retest).

Overall, these results suggest that participants specifically learned and retained both the motor and object sequences, although to a different extent as performance was better on the motor as compared to the object task (see “Discussion” and Section D in S1 Text for brain-behavior correlation analyses). Importantly, performance on the two sequence tasks was similar in the scanner and a sequence-specific performance advantage was observed despite the interleaved nature of practice.

### fMRI results

**Fig 3:**
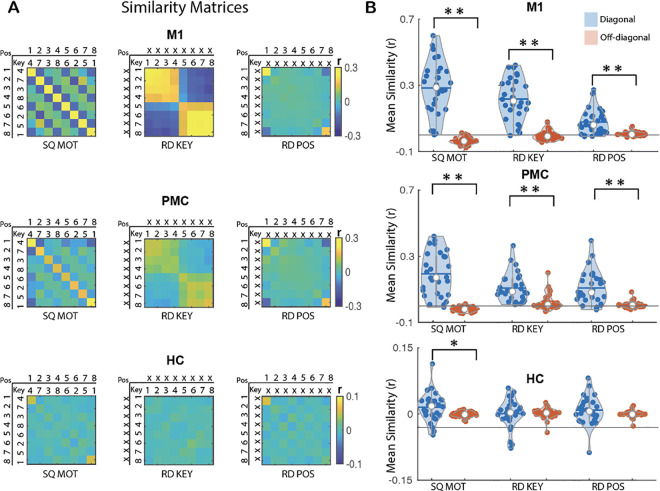
MVPA results for motor sequence learning. **(A)** Group average neural similarity matrices for the ROIs involved in motor learning. Pattern similarity was computed across repetitions of the motor sequence to assess finger-position coding in the sequence condition (SQ MOT matrix, left panel) as well as across repetitions of the random patterns to quantify finger/key (RD KEY matrix, middle panel) and position (RD POS matrix, right panel) coding in the random condition. Color bar represents mean similarity (r). In the POS rows/columns, numbers represent the temporal position in the 8-element sequence or in the random stream. In the KEY rows/columns, numbers represent fingers. (X) represents a random position or key in key and position matrices, respectively. The SQ MOT matrix represents the sequence performed by an exemplary individual (key 4 in position 1, key 7 in position 2, etc.). **(B)** Mean pattern similarity for diagonal (blue) and off-diagonal (red) cells as a function of matrices and ROIs. Results indicate that M1 and PMC show evidence of finger-position coding in the sequence condition as well as finger and position coding during random practice. In contrast, the hippocampus (HC) shows evidence for finger-position coding in the sequence condition but not for finger or position coding in the random condition. Asterisks indicate significant differences between diagonal and off-diagonal (one sided paired sample *t* test; Bonferroni corrected *p_*corr*_ < .05 and **p_*corr*_ < .001). Colored circles represent individual data, jittered in arbitrary distances on the *x*-axis to increase perceptibility. Horizontal lines represent means and white circles represent medians. The shape of the violin [23] depicts the kernel density estimate of the data. Note that as in earlier research [16], *Y* axis scales are different between cortical and subcortical ROIs to accommodate for differences in signal-to-noise ratio (and therefore in effect sizes) between ROIs. The source data corresponding to this figure are included in S1 Data, “Fig 3A” and “Fig 3B” sheets.

**Fig 4:**
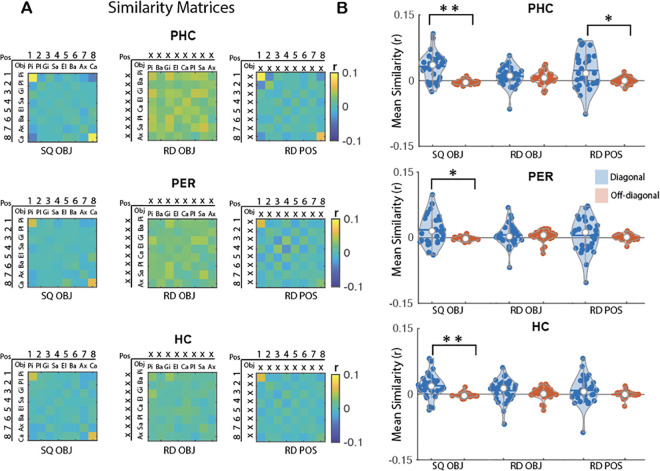
MVPA results for object sequence learning. **(A)** Group average neural similarity matrices for the 3 object ROIs. Pattern similarity was computed across repetitions of the object sequence to assess object-position coding in the sequence condition (SQ OBJ matrix, left panel) as well as across repetitions of the random patterns to quantify object coding (RD OBJ matrix, middle panel) and position coding (RD POS matrix, right panel) in the random condition. Color bar represents mean similarity (*r*). In the POS rows/columns, numbers represent the temporal position in the 8-element sequence or in the random stream. In the OBJ rows/columns, the first 2 letters of each object are presented. (X) represents a random position or object in the object and position matrices, respectively. The SQ OBJ matrix represents the sequence performed by an exemplary individual (pineapple in position 1, plane in position 2, etc.). **(B)** Mean pattern similarity for diagonal (blue) and off-diagonal (red) cells as a function of matrices and ROIs. Results indicate that the PHC shows evidence for object-position coding in the sequence condition as well as position coding during random practice. In contrast, PER and HC only show evidence for object-position coding in the sequence condition. Asterisks indicate significant differences between diagonal and off-diagonal (one sided paired sample *t* test; Bonferroni corrected *p_*corr*_ < .05 and **p_*corr*_ < .001). Colored circles represent individual data, jittered in arbitrary distances on the x-axis to increase perceptibility. Horizontal lines represent means and white circles represent medians. The shape of the violin [23] depicts the kernel density estimate of the data. The source data corresponding to this figure are included in S1 Data, “Fig 4A” and “Fig 4B” sheets.

**Fig 5:**
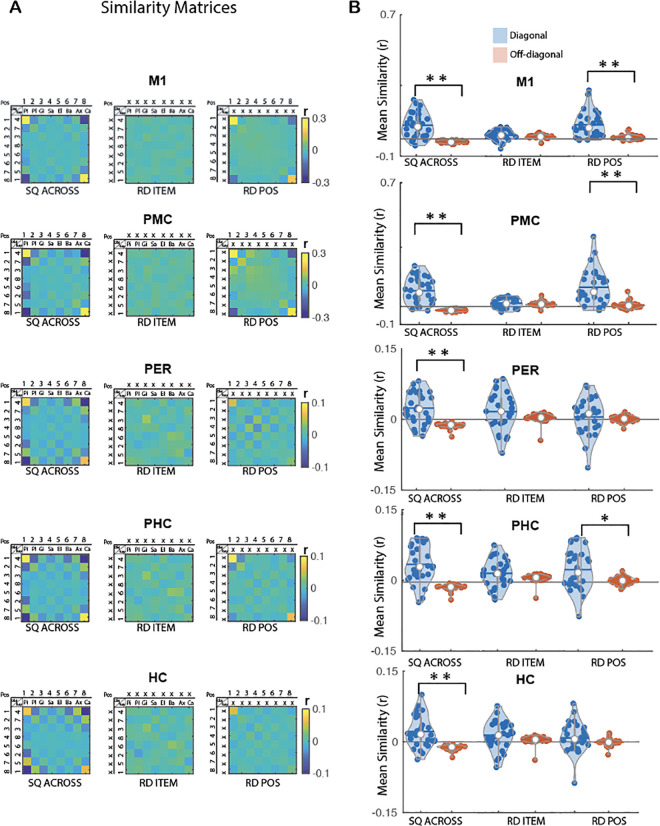
MVPA results for sequence learning across memory domains. **(A)** Group average neural similarity matrices for all 5 ROIs. Pattern similarity was computed between pairs of the individual objects from the object sequence condition and individual key presses from the motor sequence condition to assess item-position coding across sequence conditions (SQ ACROSS matrix, left panel) as well as across repetitions of the random patterns to quantify item coding (RD ITEM matrix, middle panel) and position coding (RD POS matrix, right panel) in the random condition. Color bar represents mean similarity (*r*). In the POS rows/columns, numbers represent the temporal position in the 8-element sequences or the random stream. In the KEY rows/columns, numbers represent fingers. In the OBJ rows/columns, the first 2 letters of each object are presented. (X) represents a random position or key/object in the item and position matrices, respectively. The SQ ACROSS matrix represents the sequences performed by an exemplary individual (motor sequence: key 4 in position 1, key 7 in position 2, etc. and object sequence: pineapple in position 1, plane in position 2, etc.). **(B)** Mean pattern similarity for diagonal (blue) and off-diagonal (red) cells as a function of matrices and ROIs. Results indicate that M1, PMC and PER show evidence for item-position coding in the sequence condition and position coding while HC and PER only represent item-position coding across sequences. Asterisks indicate significant differences between diagonal and off-diagonal (one sided paired sample *t* test; Bonferroni corrected *p_*corr*_ < .05 and **p_*corr*_ < .001). Colored circles represent individual data, jittered in arbitrary distances on the *x*-axis to increase perceptibility. Horizontal lines represent means and white circles represent medians. The shape of the violin [23] depicts the kernel density estimate of the data. Note that as in earlier research [16], *Y* axis scales are different between ROIs to accommodate for differences in signal-to-noise ratio (and therefore in effect sizes) between ROIs. The source data corresponding to this figure are included in S1 Data, “Fig 5A” and “Fig 5B” sheets.

The primary goal of the multivariate pattern analyses was to characterize the extent to which the ROIs *coded* for temporal order information during the execution of learned sequences. To do so, we conducted representational similarity analyses (RSA) of the fMRI data collected while participants performed the sequence learning tasks on day 2 (see Fig 1B). These analyses are based on the assumption that if a particular type of information is processed by a particular region of interest (ROI), then it will be represented in the pattern of activation across voxels in that brain region [22]. Such analyses therefore predict higher correlations in voxel activity between pairs of trials sharing this information (diagonal cells) as compared to pairs of trials not sharing this information (off-diagonal cells; see exemplary matrices depicted in Figs 3A, 4A, and 5A). This approach was used to test whether our ROIs represent information about specific items (fingers or objects, both) in their learned temporal position in the sequence within each memory domain (i.e., finger-position coding in the motor domain and object-position coding in the declarative memory domain) but also regardless of the memory domain (i.e., item-position coding across domains). To assess the specificity of this temporal order coding, we used the random task data to test for potential coding of overlapping representations (i.e., coding of the item itself and/or of temporal position itself). We therefore tested on the random data whether the ROIs carried information about (1) items irrespective of their position in the pattern (i.e., finger/object coding), and (2) temporal positions irrespective of item (i.e., position coding). Analyses were performed on five bilateral ROIs involved in sequence learning [15,16]: the primary motor cortex (M1), the premotor cortex (PMC), the perirhinal cortex (PER) the parahippocampus (PHC) and the hippocampus (HC). Results are presented below within each memory domain first and between memory domains last. Correction for multiple comparisons was performed using False Discovery Rate (FDR) correction for 5 ROIs in both the within- and between-memory domains analyses.

### Motor memory domain

Within-motor memory domain analyses first examined *selective coding* in the ROIs. This was assessed by comparing the mean correlation of the diagonal elements of the representational similarity matrices with the mean correlation of the off-diagonal elements (see Fig 3A). Specifically, we measured the extent to which specific ROIs represent information about (1) fingers in their learned temporal position in the sequence (i.e., finger-position coding in the sequence condition), (2) fingers irrespective of their position (i.e., finger coding in the random condition), and (3) temporal positions irrespective of the finger (i.e., position coding in the random condition). Next, when the ROIs presented evidence of coding for multiple representations (e.g., both finger-position and finger coding), we performed linear regression analyses to assess the *contribution of the different representations* to finger-position coding. Within-motor memory domain analyses were performed on all 5 ROIs, but only the results corresponding to the 3 ROIs involved in motor sequence learning (i.e., the primary motor cortex M1, the premotor cortex PMC and the hippocampus HC [16] are presented in the main text (see S1 and S2 Figs for results related to the other ROIs).

#### Finger-position coding in a sequence.

To investigate whether the motor sequence ROIs represent information about fingers and their learned temporal position in the sequence, we computed the correlation in voxel activity patterns between pairs of individual key presses across repetitions of the motor sequence. This resulted in an 8 × 8 motor sequence condition neural similarity matrix for each motor ROI (Fig 3A). Analysis of this matrix (***SQ MOT***) revealed significantly higher mean similarity along the diagonal (i.e., same finger + position) as compared to the off-diagonal (i.e., different finger + position) in all motor ROIs (paired sample *t* test: M1, *t*(29) = 9.72, *d* = 1.78, *p*_corr_ < 0.005; PMC, *t*(29) = 8.2, *d* = 1.5, *p*_corr_ < 0.005; HC, *t*(29)=2.47, *d* = 0.45, *p*_corr_ = 0.05, Fig 3B). These results indicate that all motor ROIs carry information about fingers in their learned temporal position in the sequence.

#### Finger and position coding in the random condition.

As each finger movement in the sequence condition was always executed at the same temporal position, any similarity effects observed in the finger-position analysis described above could be driven by the coding of finger and/or position information. Control analyses were therefore performed using the random data to test whether the motor ROIs also represented information about finger and position. To assess finger coding, we computed similarity in activation patterns between individual key presses across repetitions of the random series. The resulting 8 × 8 matri*x* (***RD KEY***, Fig 3A) revealed significantly higher mean similarity along the diagonal (i.e., same finger/key) as compared to the off-diagonal (i.e., different finger/key) in M1 and PMC (paired sample *t* test: M1, *t*(29) = 9.95, *d* = 1.82, *p*_corr_ < 0.005; PMC, *t*(29) = 7.17, *d* = 1.31, *p*_corr_ < 0.005) but not in the HC (*t*(29) = −0.19, *d* = −0.04, *p*_corr_ = 1, Fig 3B). To assess position coding irrespective of the finger, we computed similarity in activation patterns between individual positions in a sequence across repetitions of the random series. The resulting 8 × 8 matrix (***RD POS***, Fig 3A) showed significantly higher mean similarity along the diagonal (i.e., same position) as compared to the off-diagonal (i.e., different position) in M1 and PMC (paired sample *t* test: M1, *t*(29) = 5.74, *d* = 1.05, *p*_corr_ < 0.005; PMC, *t*(29) = 6.12, *d* = 1.12, *p*_corr_ < 0.005) but not in the HC (*t*(29) = 1.45, *d* = 0.26, *p*_corr_ = 0.4, Fig 4B). Importantly, similar to our earlier research [16], position coding was particularly pronounced at the edges of the stream of 8 elements (i.e., start and end of the stream – see top left and bottom right corner cells in *RD POS* matrix in Fig 3A). To verify whether position coding was driven by these boundary effects, we performed control analyses excluding boundary positions from the position matrix. These control analyses showed that position coding only remained significant in the PMC (see S1 Table for corresponding statistics) and therefore suggest that PMC carries information about position (independent of the strong boundary effects) under random conditions while position coding in M1 appears to be heavily influenced by boundary information. Altogether, these results indicate that M1 and PMC carry information about finger and position (PMC) or boundary position (M1) during random practice.

#### Contribution of finger and position coding to finger-position coding.

Given that M1 and PMC coded for finger and position/boundary information, it is possible that an overlap in finger and/or position information might contribute to the enhanced pattern similarity for finger-position coding in the learned sequence. To test for this possibility, we performed linear regression analyses to examine whether finger and position coding (as assessed during random practice) in M1 and PMC explained pattern similarity effects observed in the sequence condition. As expected, both finger and position/boundary information contributed to finger-position coding in the motor sequence in M1 (key: *B* = 0.90, *t*(_1,27_) = 6.5, *p*_*corr*_ < 0.005; pos: *B* = 1.18, *t*(_1,27_) = 4.83, *p*_*corr*_ < 0.005) and PMC (key: *B* = 0.77, *t*(_1,27_) = 4.08, *p*_*corr*_ < 0.005; pos: *B* = 1.15, *t*(_1,27_) = 9.02, *p*_*corr*_ < 0.005, and see S2 Table for results on all other ROIs).

#### Summary.

Altogether, these results indicate that hippocampal patterns carry information about fingers in their learned temporal position in the motor sequence condition (i.e., finger-position coding). Although M1 and PMC showed evidence of finger-position representation in the sequence condition, these regions also exhibited finger and position (PMC) or boundary position (M1) coding in the random condition. Linear regressions indicate that finger-position coding in M1 and PMC during the execution of the learned sequence can, at least in part, be attributed to finger and position/boundary coding.

### Declarative memory domain

As above, within-declarative memory domain analyses first examined *selective coding* in the ROIs. We measured here the extent to which specific ROIs represent information about (1) objects in their learned temporal position in the sequence (i.e., object-position coding in the sequence condition), (2) objects irrespective of their position (i.e., object coding in the random condition), and (3) temporal positions irrespective of the object (i.e., position coding in the random condition). When the ROIs presented evidence of coding for multiple representations (e.g., both object-position and object coding), we performed linear regression analyses to assess the *contribution of the different representations* to object-position coding. Within-declarative memory domain analyses were performed and corrected on all 5 ROIs but only results corresponding to the 3 ROIs involved in object sequence learning (i.e., the perirhinal cortex PER, the parahippocampus PHC and the hippocampus HC) are presented in the main text (see S1 and S2 Figs for results related to the other ROIs).

#### Object-position coding in a sequence.

To investigate whether the object sequence ROIs represent information about objects in their learned temporal position in the sequence, we computed the correlation in voxel activation patterns between pairs of individual objects across repetitions of the object sequence (Fig 4A). The resulting 8 × 8 matrix (***SQ OBJ***, Fig 4A) revealed significantly higher mean similarity along the diagonal (i.e., same object + position) as compared to the off-diagonal (i.e., different object + position) in all object ROIs (paired sample *t* test: PHC, *t*(29) = 5.62, *d* = 1.03, *p*_*corr*_ < 0.005; PER, *t*(29) = 2.95, *d* = 0.54, *p*_*corr*_ = 0.02; HC, *t*(29) = 3.73, *d* = 0.68, *p*_*corr*_ < 0.005, Fig 4B). These results indicate that all object ROIs carry information about objects in their learned temporal position in a sequence.

#### Object and position coding in the random condition.

Similar to above, control analyses were performed using the random data to test whether the ROIs also represented information about object and position. Results derived from the 8 × 8 ***RD OBJ*** matrix (Fig 4A) did not reveal any significant differences in mean similarity along the diagonal (i.e., same object) as compared to the off diagonal (i.e., different object) in any of the object ROIs (PHC, *t*(29) = 1.06, *d* = 0.19, *p*_*corr*_ = 0.74; PER, *t*(29) = 0.54, *d* = 0.1, *p*_*corr*_ = 1; HC, *t*(29) = 1.49, *d* = 0.27, *p*_*corr*_ = 0.37, Fig 4B). The 8 × 8 ***RD POS*** matrix (Fig 4A) revealed significantly higher mean similarity along the diagonal (i.e., same position) as compared to the off-diagonal (i.e., different position) for the PHC (*t*(29) = 2.88, *d* = 0.53, *p*_*corr*_ = 0.02) but not for the PER or the HC (PER, *t*(29) = 0.56, *d* = 0.1, *p*_*corr*_ = 1; HPC, *t*(29) = 1.45, *d* = 0.26, *p*_*corr*_ = 0.4). It is worth noting that position coding in the PHC was no longer significant after removing boundary positions from the position matrix (see S1 Table for corresponding statistics). These results suggest that PHC carries information about boundary positions.

#### Contribution of position coding to object-position coding.

As the PHC presented evidence of object-position coding and boundary position coding, we performed linear regression analyses to examine whether boundary position coding (as assessed during random practice) might explain pattern similarity effects observed in the sequence condition. As expected, boundary position information contributed to the object-position coding effect in the object sequence in the PHC (*B* = 0.49, *t*(_1,28_) = 4.28, *p*_*corr*_ < 0.005 and see S3 Table for results in all other ROIs).

#### Summary.

Overall, these results indicate that HC and PER patterns carry information about objects in their learned temporal position in the object sequence condition (i.e., object-position coding). Although PHC showed evidence of object-position coding in the sequence condition, this region also exhibited position (edge) coding in the random condition. Linear regression analyses indicated that object-position coding in the PHC during the execution of the learned sequence can, at least in part, be attributed to position/boundary coding.

### Domain-general effects (across memory domains)

As above, between-memory domains analyses first examined *selective coding* in the ROIs. We measured here the extent to which specific ROIs represent information about (1) items (irrespective of their nature) in their learned temporal position in the sequence (i.e., item-position coding in the sequence condition), (2) items irrespective of their nature and their position (i.e., item coding in the random condition), and (3) temporal positions irrespective of the item (i.e., position coding in the random condition). When the ROIs presented evidence of coding for multiple representations (e.g., both item-position and position coding), we performed linear regression analyses to assess the *contribution of the different representations* to item-position coding across domains.

#### Item-position coding across sequences of different domains.

We tested whether the representation of temporal order information during the execution of learned sequences is content-free. To do so, we examined whether items from different domains (i.e., fingers and objects) but sharing the same temporal position in the different sequences (e.g., key X in position 2 in the motor sequence and object Y in position 2 in the object sequence) elicited similar multivoxel brain patterns. We therefore build a representational similarity matrix in which voxel activation patterns were correlated between the object and motor sequence tasks. The resulting 8 × 8 matrix (***SQ ACROSS***, Fig 5A) revealed significantly higher mean similarity along the diagonal (i.e., object/key + same position) as compared to the off-diagonal (i.e., object/key + different position) in all ROIs (paired sample *t* test: M1, *t*(29) = 6.97, *d* = 1.27, *p*_*corr*_ < 0.005; PMC, *t*(29) = 7.96, *d* = 1.45, *p*_*corr*_ < 0.005; HC, *t*(29) = 5.61, *d* = 1.02, *p*_*corr*_ < 0.005; PHC, *t*(29) = 6.76, *d* = 1.23, *p*_*corr*_ < 0.005; PER, *t*(29) = 6.26, *d* = 1.14, *p*_*corr*_ < 0.005, Fig 5B). These results indicate that all ROIs carry information about items (irrespective of their domain) in their learned position in the sequence.

#### Item and position coding in the random condition.

Similar as above, control analyses were performed using the random data to test whether the ROIs also represented information about item across domains (i.e., similarity between, e.g., key X and object Y that were presented in the same temporal position in the sequence condition) and position information (as above). Results derived from the ***RD ITEM*** 8 × 8 matrix (Fig 5A) did not reveal any significant differences in mean similarity between the diagonal (i.e., object and key in the random condition but that were presented in the same temporal position in sequence condition) as compared to the off diagonal (i.e., objects and keys with different temporal positions in the sequence) in any of the ROIs (M1, *t*(29) = 1.04, *d* = 0.19, *p*_*corr*_ = 0.77; PMC, *t*(29) = 0.52, *d* = 0.1, *p*_*corr*_ = 1; HC, *t*(29) = 1.64, *d* = 0.3, *p*_*corr*_ = 0.28; PHC, *t*(29) = 1.86, *d* = 0.34, *p*_*corr*_ = 0.19; PER, *t*(29) = 1.94, *d* = 0.35, *p*_*corr*_ = 0.16, Fig 5B). As a reminder, position coding results described above (8 × 8 ***RD POS***, Fig 5A) indicate that PMC carries information about position in random patterns and that both M1 and PHC represent information about boundary position in random patterns.

#### Contribution of position coding to item-position coding across sequences of different domains.

Given that M1, PMC and PHC presented evidence of item-position coding across sequences of different domains and also coded for position/boundary, we performed linear regression analyses to examine whether the position/boundary coding (as assessed during random practice) in these ROIs might explain pattern similarity effects observed in the sequence condition across memory domains. As expected, position/boundary information contributed to the item-position coding across memory domains in these ROIs (M1, B = 0.89, *t*(_1,28_) = 7.37, *p*_*corr*_ < 0.005; PMC, *B* = 0.72, *t*(_1,28_) = 9.39, *p*_*corr*_ < 0.005; PHC, *B* = 0.51, *t*(_1,28_) = 3.75, *p*_*corr*_ < 0.005, see S4 Table for results on all other ROIs).

#### Summary.

Overall, these results indicate that HC and PER multivoxel patterns carry information about items – irrespective of their memory domain – in their learned temporal position in the sequence condition (i.e., item-position coding). In other words, the patterns associated to a finger are similar to those associated to an object if these items are presented in the same temporal position in a learned sequence. Although M1, PMC and PHC showed evidence of item-position coding across sequences from different domains, these regions also exhibited position (PMC) or boundary position (M1 and PHC) coding in the random condition. Linear regression analyses indicated that item-position coding in these regions during the execution of the learned sequence can, at least in part, be attributed to position/boundary coding.

## Discussion

In the current study, we used multivoxel representational similarity analyses of fMRI data acquired during the execution of learned sequences from different memory domains to test whether the brain processes supporting temporal order coding of sequential behaviors are domain-specific or – general. Our results indicate that the hippocampus and the perirhinal cortex represent information about items in their learned temporal position within both memory domains separately, but also across memory domains, i.e., regardless of whether the item in a particular position is a finger or an object. These regions did not show evidence of finger/object, item or position coding during random practice. In contrast, in all other ROIs, pattern similarity results in the sequence condition within and between memory domains were, at least in part, explained by finger and/or (boundary) position coding assessed in the random condition. Specifically, multivoxel patterns in the primary motor cortex and the premotor cortex represented both finger- and (boundary) position-based information during random practice while the parahippocampus carried information about boundary positions.

Our results indicate that hippocampal multivoxel patterns carried information about the position of items in a learned sequence within both memory domains but also across sequences from different memory domains. These results suggest that the hippocampus similarly encodes the temporal order of items in a sequence irrespective of their nature. Importantly, and in contrast to other ROIs (but see “Discussion” on the perirhinal cortex below), the hippocampus did not represent information about finger/object, item or position during random practice. The observation of item-position coding in a learned sequence within each memory domain is consistent with earlier research. In the motor sequence learning domain, our results are in line with our previous multivariate fMRI research demonstrating that the hippocampus represents information about the temporal position of movements during the execution of learned motor sequences but not about finger or temporal position in random patterns [16]. They also concur with previous fMRI and MEG research indicating that the hippocampus also represents temporal information of learned motor sequences *offline*, i.e., during both planning (parahippocampus [24], hippocampus [25]) and reactivation [11,12] periods preceding and following motor sequence practice, respectively. In the object sequence learning domain, our data concur with a previous study using a similar deterministic object sequence task as in the current study [15]. As above, this work showed hippocampal representations of the temporal position of objects in a sequence but not for object or temporal position in random patterns. Interestingly, our findings are also consistent with previous research suggesting that hippocampal multivoxel patterns are modulated by temporal regularities in a series of objects [26]. It is worth noting though that it is difficult to draw parallels between the current work and this earlier study as the object sequence learning paradigms were different in nature. Specifically, this previous study employed a probabilistic object sequence learning task that required statistical learning as opposed to the explicit deterministic sequence learning in the current manuscript. Additionally, our object sequence task presumably necessitated more explicit, strategy-based processes to support the complex set of computations required for the visual search necessary to map the series of objects to responses on the keyboard. Nevertheless, it is tempting to speculate that the process of representing the temporal structure of elements in a stream being similar across both object sequence tasks, it might not depend on the nature of the sequential task *per se* (i.e., explicit deterministic versus implicit statistical learning, strategy-based versus passive learning, etc.).

Altogether, our results are consistent with evidence suggesting that the hippocampus processes temporal order information during sequence learning irrespective of the nature of the items (e.g., letters, odors, objects and movements; [15,16,18,27] and the type of memory (i.e., motor versus non-motor: [15,16]; spatial versus non-spatial: [28,29]). Importantly, we show here that hippocampal multivoxel patterns are similar between items from different domains (i.e., pattern associated to a movement versus an object) when they are presented in the same ordinal position in a learned sequence. This is in line with earlier views proposing that the hippocampus represents the associative map linking response goals – rather than item-specific information – to their ordinal position in a sequence [16]. The current data suggests that such map, abstracted during sequence learning, is independent of the type of learned items to order and might serve as a cognitive spatiotemporal framework to order sequential behaviors irrespective of their domains. This concurs with the sequence-generator hypothesis, an influential model of hippocampal functioning suggesting that the hippocampus provides a sequential, content-free, structure that preserves the order of experiences irrespective of their nature [19]. Altogether, these results provide the first experimental evidence that the capacity of the hippocampus to encode temporal order during sequence learning is a general process that is shared across memory domains.

Albeit unexpected, a similar pattern of results was observed in the perirhinal cortex. Specifically, perirhinal multivoxel patterns of activity represented information about items in their learned temporal position in an object sequence, in a motor sequence (see S1 and S2 Figs) and regardless of the type of sequence while no item nor position coding was observed. This contradicts earlier research showing that the perirhinal cortex represents information about objects [15,30,31] but not about objects in their learned temporal position in a sequence [15]. The reason for these discrepancies is unclear but one could argue that they are related to the visual stimulation paradigm used in our experiment. Our task necessitated to not only present the central object (visual cue triggering movement) on the screen but also to constantly display the object-key mapping at the bottom of the screen. This resulted in the simultaneous presentation of multiple objects on the screen which may have masked the representation of the central cued objects in the perirhinal cortex. While this is possible, we argue that this is unlikely as we were able to observe the well-known object-category representation in the ventral occipito-temporal cortex (e.g., [32,33], see S3 Fig) which suggests successful visual processing of the central image in this brain area. Based on these results, we cannot completely rule out that object coding did not contribute to item-position coding in the perirhinal cortex but the current pattern of results suggest that, similar to the hippocampus, the perirhinal cortex is specifically involved in associating memory items to their learned position in a sequence regardless of the nature of the learned item. These findings are in contrast with earlier work suggesting that the perirhinal cortex is involved in representing *domain-specific* relational binding information that is fed into the hippocampus for *domain-general* processing [34]. However, they are line with a growing body of literature showing *domain-general* effects in the perirhinal cortex for the encoding of relationships between items and events [35], including item-item [36], item-time [37] and item-location [38] associations. We therefore propose that both the perirhinal cortex and the hippocampus have a similar capacity to represent memory items in their learned temporal position in a sequence, irrespective of the nature of the items to order.

In contrast to the medial temporal lobe regions discussed above, the primary motor cortex, the premotor cortex and the parahippocampus carried information about multiple representations (i.e., item-position and item/position coding). The results of the linear regression analyses indicated that the contribution of these brain regions to sequence learning across domains is attributed – at least in part – to the coding of some of these overlapping representations. In the premotor cortex, position coding significantly contributed to item-position coding across sequences of different domains. The role of the premotor cortex in temporal processing is well described in the motor domain. For example, animal work has shown that premotor cortex neurons are tuned to specific positions in a series of movements [39] and multivariate analyses of human fMRI data indicate that the premotor cortex encodes temporal features about repetitive movements irrespective of their patterns (different sequences, [40]; sequence and random patterns, [16]). The premotor cortex is therefore thought to provide a temporal scaffold in which movements are implemented during motor task practice [16]. We are not aware of any report of such function of the premotor cortex in the declarative memory domain (but note that the premotor cortex is usually not considered a region of interest in this memory domain). We therefore argue that the involvement of the premotor cortex in position coding across domains in the current study (as well as within each memory domain, see S2 Fig and S2–S4 Tables) is related to the motoric nature of the task rather than to a temporal processing function shared across memory domains. Indeed, all task variants (including the object sequence task) required participant to respond to each cue with a key press. In line with earlier work [16,40], we suggest that the involvement of the premotor cortex is therefore presumably related to the implementation of a temporal scaffold in which movements are implemented irrespective of the version (motor versus object) and sequential nature (sequence versus random, respectively) of the task. This, however, remains speculative.

In the primary motor cortex, boundary position coding significantly contributed to item-position coding across domains. The boundary effects observed in this study are consistent with our earlier research showing greater similarity values at the start and end of the stream of 8 elements (in both motor sequence and random conditions) across multiple regions of interest including the primary motor cortex [16]. It is worth noting that these boundary effects are particularly pronounced in our study in comparison to our earlier work [16]. We hypothesize that this is related to the change in object/key mapping that occurred after each stream of 8-elements and that was designed to orthogonalize motor and object sequence tasks in the current study. This might have accentuated attentional [41] and anticipatory [42] processes in the primary motor cortex; processes that are known to increase the similarity of brain responses [43]. We therefore suggest that the contribution of the primary motor cortex to item-position coding across memory domains is related to non-memory related attentional processes that were particularly pronounced in this paradigm.

Similar to the primary motor cortex, boundary position coding in the parahippocampus significantly contributed to item-position coding across memory domains. Earlier research has shown that the parahippocampus codes for the temporal position of objects presented in a stream irrespective of whether they are presented in a sequential or random order [15]. It is also suggested that the parahippocampus carries information about the context in which objects are encountered [44,45]. Previous MEG research also indicated such temporal function in the parahippocampus during motor learning and showed that this region is involved in coding an abstract template of ordinal position during motor sequence preparation [24]. Together with the evidence reviewed above demonstrating parahippocampal involvement in temporal coding in both the declarative and the procedural memory domains, the current findings suggest that the role of the parahippocampus in position coding might be shared across memory domains. However, it is important to note that, similar to the primary motor cortex, position coding in our dataset was no longer observed in the parahippocampus after removing boundary positions. It is therefore possible that similar attentional processes as discussed above might have contributed to the pattern of results in the parahippocampus. Altogether, while our data and earlier research both point to a similar position coding effect between memory domains in the parahippocampus, it cannot be ruled out that these effects are related to boundary positions only.

Last, the current within-motor memory domain results partly confirm our earlier research [16]. Specifically, our data indicate that the contribution of motor cortical regions to motor sequence learning is attributed – at least in part – to movement and position coding. Specifically, in the primary motor cortex, both finger and boundary position information contributed to finger-position coding. The contribution of finger information to finger-position coding is consistent with earlier multivariate fMRI studies demonstrating that although the primary motor cortex receives input from areas showing motor sequence representation, multivoxel activity patterns in this region relate to the execution of individual key presses during motor sequence learning [16,46]. The contribution of boundary position coding to finger-position coding in the primary motor cortex was not observed in our earlier research [16] and might be attributed to the nature of the current paradigm (see above). In the premotor cortex, both finger and position (beyond boundaries) information contributed to finger-position coding. The observation of finger coding in the premotor cortex contradicts earlier research indicating that this region encodes sequential context rather than individual movement components [46–48]. However, they are in line with our recent work [16] suggesting that the premotor cortex represents finger information during early stages of learning (as in the current study) while sequence representations may emerge at later learning stages [46,48]. As discussed above, our results point to a role of the premotor cortex in temporal position coding which is in line with both previous animal [39] and human [16,40] research and speak to the capacity of the premotor cortex to provide a temporal scaffold in which movements are implemented.

A limitation in our study is that, except for the MRI session, participants performed significantly better in the motor sequence task than in the object sequence task. We speculate that these differences are related to the nature of the different tasks and do therefore not necessarily reflect difference in learning amplitude between tasks. Specifically, as learning progressed in the motor version of the task, participants might have been able to press the sequence of keys without relying on the key-object mapping displayed on the screen. In contrast, the object sequence task required participants to use the key/object mapping to respond even at later stages of learning, which might have slowed performance down. This hypothesis is supported by the data from the MRI sessions that were acquired with longer response-stimulus-interval (see “Materials and methods”) – which afforded additional time for the mapping that was particularly beneficial for the object task – and during which similar performance was observed between motor and object sequence conditions. As earlier research suggests that performance speed can alter brain representations [47], we performed additional control analyses to assess potential relationships between performance and brain patterns. Specifically, we tested whether the level of performance – and potentially the strength of the memory trace – reached at the end of training on day 1 was related to the strength of the motor and object sequence representations assessed during the MRI session the next day (see Section D in S1 text). Our results showed no correlation between performance and finger/object-position coding in any of the ROIs, suggesting that the difference in performance observed between tasks on day 1 is unlikely to influence the pattern of brain results reported above. Another limitation in the current design, is that it does not allow us to determine the extent to which the perceptual component (i.e., statistically-consistent patterns of visuospatial orienting from the central cue to the spatial location of the corresponding finger) of the motor task contributed to our findings in the hippocampus for the motor condition. It is worth noting however that previous studies have reported significant hippocampal activity during motor sequence learning even when the task did not include any visual guidance (e.g., [9,49–54]) and in visually-guided tasks where perceptual learning was controlled (e.g., [8,55]). These prior studies suggest that the involvement of the hippocampus in motor sequence learning is not limited to motor tasks presenting perceptual attributes. We would therefore expect similar results as those reported in the current manuscript with a non-visually guided motor task, provided that the task requires to preserve the sequential order of movements. Additionally, as the statistically-consistent patterns of visuospatial orienting was not present in the object sequence task (as key presses were random, the visuospatial orienting from the central cue to the spatial location of the corresponding finger did not have a consistent pattern) and this process was therefore not shared across object and motor tasks, we argue it is unlikely that hippocampal activation patterns *across* the two tasks represent processes that are not shared across tasks. Although we cannot rule out that hippocampal patterns across the two sequential tasks reflect different processes or features within the visual domain (i.e., visuo-spatial learning in the motor sequence task and object sequence learning in the object sequence task), we rather argue that hippocampal patterns that are similar across the two sequential tasks reflect processes that are shared across tasks. Specifically, we propose that the hippocampus represents the temporal order of learned items in a sequence rather than different task features within the same (visual) domain. However, this remains speculative and warrants further examination.

To conclude, our findings suggest that medial temporal lobe regions such as the hippocampus and the perirhinal cortex play a critical role in representing memory items – irrespective of their domains – in their learned temporal positions in a sequence. We propose that these regions contribute to the development of memory-domain general item-position maps that provide a content-free cognitive framework to order sequential behaviors irrespective of their nature. In line with the sequence-generator hypothesis [19], our results support the view that the medial temporal lobe provides a series of indices that point to cortical modules carrying content-specific information (e.g., primary motor cortex for finger information and premotor cortex for position information). Our findings concur with the hypothesis that these different task-specific inputs are sequenced by activity patterns in the medial temporal lobe, thus preserving the ordinal structure over which experiences occur, irrespective of their nature.

## Materials and methods

### Participants

Sample size computation was done with G*Power [56]. The effect size was calculated based on previous work on motor memory [16] that detected significant differences in hippocampal activation patterns between conditions within the motor domain (i.e., 3 conditions: finger-position, finger and position information; main effect of condition; effect size = 0.40). The correlation coefficient was set to 0.5 for a more conservative power calculation to account for the different nature of the present design (2 tasks; 6 conditions). Sphericity correction was set to 0.5 to account for potential deviation from the sphericity assumption. Alpha was set to 0.01 to correct for the number of ROIs (5) and a power of 0.8. The required sample size was 28 participants.

To account for attrition, 33 young healthy adults aged 20–35 (18 female, mean age = 26.3, SD = 5.2) were recruited to participate in this study. All participants completed an online screening questionnaire to verify their eligibility to take part in the study. All participants were right-handed (Edinburgh Handedness Questionnaire; [57], self-reported no current or previous history of neurological, psychological, or psychiatric conditions, and were free of any psychotropic or sleep impacting medications. None of the participants received previous extensive training as a musician or as a professional typist. They also presented no contra-indication to MRI. Further screening using standardized questionnaires also ensured that participants did not exhibit any indication of anxiety (Beck Anxiety Inventory (BAI); [58]) or depression (Beck Depression Inventory (BDI); [59]). Participants reported normal sleep quality and quantity during the month prior to the study (Pittsburgh Sleep Quality Index (PSQI); [60]) as well as normal levels of daytime sleepiness (Epworth Sleepiness Scale; [61]). None of the participants were extreme evening or morning type individuals (Morningness–Eveningness Questionnaire (MEQ); [62]). A total of 30 participants were included in the analysis. One participant was excluded for low performance accuracy on the sequence tasks (below the mean – 3 standard deviations from the group), another for excessive movement in the MRI scanner across multiple runs (>2 voxels, see below for movement information at the group level) and another for a non-compliant sleep schedule between experimental sessions (slept less than 7h and went to sleep later than 1am). All study procedures were approved by the University of Utah institutional review board (IRB_00155080). All participants provided written informed consent at the start of the study and received monetary compensation for their time. The study was conducted according to the principles expressed in the Declaration of Helsinki. Participants’ demographics as well as information on their sleep and vigilance are presented in S5 Table.

### Serial reaction time task (SRTT)

All participants performed an adapted version of the explicit Serial Reaction Time Task (SRTT; [3] which was coded in MATLAB (Mathworks , Sherbom, MA) using Psychophysics Toolbox version 3 [63]. The task consisted of an 8-choice reaction time task in which participants were instructed to react to visual cues shown on a screen by pressing a corresponding key on a keyboard (behavioral sessions) or on an MRI-compatible button box (MRI session). The large visual cue presented in the middle of the screen corresponded to one of eight different images of objects (Fig 1A, left panel). The 8 different objects were constantly presented on the bottom of the screen during task practice and mapped to each of the eight fingers used to perform the task (i.e., no thumbs). The images of the 8 objects were derived from an open access online database at http://olivalab.mit.edu/MM/uniqueObjects.html [21]. Participants were instructed to respond to the central visual cue as fast and as accurately as possible by pressing the corresponding key according to the object/key mapping displayed at the bottom of the screen (see Fig 1A, left panel). When the response was recorded, the central cue turned to a green fixation cross while the mapping with the 8 objects remained on the bottom of the screen during the full duration of the response-stimulus-interval (RSI). RSI was 750 ms for the behavioral version of the task and jittered between 1.5 and 2.5 s for the MR version of the task in order to optimize the design for MVPA (see “Procedure” and MVPA sections below). Blocks of task practice were alternated with 10 s rest periods which were indicated by a red fixation cross in the center of the screen (replacing the central cue object/green cross displayed during practice) and red frames replaced the locations of the 8 objects that displayed the key-object mapping on the bottom of the screen. Participants were made aware that the order of objects/movements would follow a repeating sequential pattern but were not told the precise sequence or the number of elements.

Importantly, the key-object mapping displayed on the bottom of the screen changed after each stream of 8 elements (i.e., every 8 objects/8 key presses) in order to orthogonalize motor and object sequence learning tasks. With this manipulation, we were able to develop three different versions of the task. In the *object sequence condition*, the stream of objects on the screen followed a sequential pattern (deterministic sequence of 8 objects, e.g., pineapple, plane, giraffe, saw, elephant, banana, axe, car) while the responses on the keyboard followed a random order (motor random). In the *motor sequence condition*, the responses on the keyboard followed a repetitive sequential pattern (deterministic sequence of 8 key presses, e.g., 4, 7, 3, 8, 6, 2, 5, 1, whereby 1 and 8 represent the left and right little fingers, respectively) while the stream of objects on the screen followed a random order (object random). In the *random task condition*, the series of both responses and objects followed a random order (i.e., motor random/object random). This experimental manipulation allowed us to examine sequence learning in the two different memory domains using the same task. Specifically, performance improvement on the motor sequence learning task (as compared to random) reflects sequence-specific skill acquisition and performance improvement on the object sequence learning task (as compared to random) reflects the acquisition of the knowledge of the order of the series of objects. As declarative memory is also defined as the conscious recollection of previous events, we conducted – on an independent sample of participants – a control behavioral experiment (see details in the “Supplementary control analyses and experiments” section below) in which we implemented an object generation/recall test similar as in earlier research and aiming at reconstructing the temporal order of the object items [15]. For completeness, a motor generation task [52] was also implemented in the control experiment to test for any explicit knowledge of the learned sequences of movements. The methods and results corresponding to this control experiment are presented in the supplemental information (Section C in S1 Text and S4 Fig).

Note that all participants learned the same object and motor sequences. However, to optimize the MVPA of the fMRI data, the starting point of the sequence in each task condition was different across participants such that the same key/object was not associated to the same temporal position in a sequence across individuals (i.e., in participant 1, the sequences started with 4/pineapple, in participant 2, the sequences started with 7/plane, etc.). The random task variant served 3 functions. The first was to ensure that general motor execution, independent of sequence learning *per se* (e.g., baseline performance levels) did not differ between the different experimental sessions. The second was to assess sequence-specific learning on Day 2 (i.e., comparison between sequence and random conditions, see above). The third was to serve as a neuroimaging control condition that allowed us to investigate which brain areas coded for temporal position (irrespective of the memory type) but also for specific task items (i.e., finger- and object-coding, see MVPA section below). In all task variants, the number and timing of key presses was recorded and used to compute behavioral outcomes (see below).

### Procedure

The experimental design is presented in Fig 1B. Participants were asked to complete 3 experimental sessions spread over 2 consecutive days. They were instructed to follow a constant sleep schedule (according to their own schedule ±1 h; latest bedtime: 1 AM; minimum 7 h of sleep; no naps) starting 3 days before the first session and between the two experimental days. Compliance to the sleep schedule was monitored using sleep diaries and wrist actigraphy (ActiGraph wGT3X-BT, Pensacola, FL). On day 1, participants learned the motor and object sequence tasks during 2 separate 1 h sessions (order counterbalanced across participants) performed outside the MRI scanner. The first session was completed in the morning (between 9 AM and 12 PM) while the second session was performed at least 4 h later (i.e., between 1 and 4 PM) in order to reduce interference between the 2 learning episodes [64]). During session 1, participants completed a brief questionnaire to collect information regarding their sleep patterns in the previous 24 h (St Mary’s Hospital Sleep Questionnaire (SMS); [65]), their subjective feelings of alertness (Stanford Sleepiness Scale (SSS); [66]) and any consumption of alcohol and/or recreational drugs in the last 24 h. Next, participants performed a psychomotor vigilance test (PVT) to obtain an objective measure of their alertness. For the PVT, participants were asked to fixate on a cross in the center of the screen and to press the space bar as fast as possible when the fixation cross disappeared. A timer appeared on the screen displaying the participants’ reaction time in milliseconds. Participants completed 50 trials of the PVT task. Next, participants were instructed to practice the SRTT task with an RSI of 750 ms (behavioral version of the task). Practice was organized in blocks of 5 repetitions of an 8-element series. First, participants completed 4 blocks of the random condition to get familiar with the task apparatus and measure baseline performance. They were then trained on 20 blocks of either the object or motor sequence condition. After a short break offered to minimize the confounding effect of fatigue on end-training performance [67], performance was tested with 4 additional blocks of practice.

After completing session 1, participants were given actigraph monitors to be worn until they returned for the final session on day 2. For the 4 h between sessions 1 and 2, participants were instructed to not take a nap or practice the task and to avoid intense exercise and consumption of stimulating substances (e.g., alcohol, caffeine, energy drinks). Session 2 followed the same procedure as session 1 except that participants were trained on the other sequential condition (object or motor). After completing session 2, participants were instructed to have a regular night of sleep according to the schedule followed prior to the experiment, to continue wearing the actigraph, to not practice the task and avoid consumption of stimulating substances. Participants returned 24 h later, at the MRI facility, for the third and final session.

On experimental day 2, for session 3, participants were asked to complete the same questionnaires that were completed on day 1 along with an MRI screening form. Next, they performed the PVT followed by 1 practice block of familiarization to the MR version of the SRTT task optimized for MVPA (slower RSI; mixed conditions; different rest pattern; see below) outside the scanner. Participants were then placed in the MR scanner so that their brain activity was recorded while performing the task on an MRI-compatible keyboard (Psychology Software Tools, Celeritas). The MRI session consisted of 8 runs (Fig 1B). Each run lasted approximately 6 min and included the 3 following conditions of interest for MVPA: motor sequence, object sequence and random. Each run comprised 5 repetitions of each condition (8 runs × 5 repetitions: 40 repetitions per condition in total) and 3 rest blocks of 10 s (to minimize fatigue) that were randomly positioned between conditions. The order of the conditions was pseudo-randomized within each run so that the same condition was not repeated more than two times in a row. The inter-stimulus-interval was jittered between 1,500 and 2500 ms. There was no additional delay between conditions. After completing the MR session, participants were tested with 4 additional blocks of each of the 2 sequential conditions followed by a final 4 blocks of the random condition outside the scanner with the behavioral version of the task (see above) to assess motor memory retention.

### fMRI data acquisition

Images were acquired with a Siemens Vida 3.0T MRI System and a 64-channel head coil. Functional images were acquired with a T2* gradient echo-planar sequence using axial slices angled to maximize whole brain coverage (*TR* = 1.03 s, *TE* = 31 ms, *FA* = 65°, 56 slices, 2.5 mm isotropic voxels, 0 mm slice gap, *FoV* = 210 × 210 mm^2^, matrix size = 88 × 88 × 56 slices, *MB* = 4). A structural T1-weighted 3D MP-RAGE sequence (*TR* = 2.5 s, *TE* = 2.98 ms, *FA* = 8°, 176 slices, *FoV* = 250 × 250 mm^2^, matrix size = 256 × 256 × 176) was also obtained for each participant during the same scanning session.

### Data analysis

#### Behavioral data.

Two measures of performance were extracted from the behavioral data. The measure of performance speed was response time (in seconds) calculated as the time between the onset of display of the central cue and the response of the participant on the keyboard. Response time on correct trials were averaged within each practice block. The measure of performance accuracy consisted of percentage of correct responses per block. To assess whether baseline performance differed between sessions on day 1, two separate repeated-measures ANOVA were conducted on performance speed and accuracy on the random SRTT data using session condition (2: object session, motor session) and block (4) as the within-subject factors (see Fig 2 and corresponding results reported in Section A in S1 Text). To assess learning over practice blocks and compare learning across conditions on day 1, separate two-way repeated-measures ANOVAs were performed on both speed and accuracy measures using condition (2: object, motor) and block (20 for training, 4 for test) as the within-subject factors. Behavioral data collected on day 2 were analyzed with separate repeated-measures ANOVA using condition (3: object, motor, random) and block (8 for the MRI session and 4 for the retest session) as the within-subject factors. Last, additional control analyses were performed to assess the effect of order on performance during learning on day 1 and retest on day 2. To do so, the analyses described above were repeated using session order, irrespective of the task condition (2: Session 1, Session 2) and block as the within-subject factors. The results corresponding to these analyses are reported in Section B in S1 Text. Statistical tests yielding *p* values ˂ 0.05 were considered significant.

#### fMRI data.

***Preprocessing. ***Anatomical and functional images were preprocessed using Statistical Parametric Mapping SPM12 (Welcome Department of Imaging Neuroscience, London, UK) implemented in MATLAB. Preprocessing included segmentation of the anatomical image into gray matter, white matter, cerebrospinal fluid (CSF), bone, soft tissue and background. Functional images were slice time corrected (reference: middle slice) and realigned using rigid body transformations, iteratively optimized to minimize the residual sum of squares between each functional image and the first image of each session separately in a first step and with the across-run mean functional image in a second step (mean translation in the three dimensions across the 8 runs: 0.27 mm (±0.11 mm), mean maximum translation in the three dimensions across the 8 runs: 0.91 mm (±0.45 mm). Movement was considered as excessive when translations exceeded more than 2 voxels in either of the three dimensions for any of the 8 runs. One individual was excluded from data analyses as such excessive movements were observed in more than half of the runs. In two other individuals, excessive movement was observed in one specific run and data could be included after truncating volumes in these runs. Specifically, in one individual, the last 111 functional images (out of 382) of one run were excluded and, in another run, the first 13 out of 387 functional images were excluded. The realigned functional images were then co-registered to the structural T1-image using rigid body transformation optimized to maximize the normalized mutual information between the two images. All analyses were performed in native space to optimally accommodate interindividual variability in the topography of memory representations [32].

##### Regions of interest.

The goal of the fMRI analyses was to examine brain patterns elicited in specific regions of interest (ROIs) by sequence and random task practice. The five following (bilateral) ROIs were selected *a priori* based on previous literature describing their critical involvement in motor and object sequence learning [15,16]: the primary motor cortex (M1) and the perirhinal cortex (PER) for item coding within the motor and declarative memory domains, i.e., for finger and object coding, respectively; the premotor cortex (PMC) and the parahippocampus (PHC) for position coding described in motor and object sequence learning, respectively; and the hippocampus (HC) for item-position coding in both domains. As in previous studies, all ROIs were anatomically defined (e.g., [16,68]). The bilateral hippocampal ROI was defined in native space using FSL’s automated subcortical segmentation protocol (FIRST; FMRIB Software Library, Oxford Centre for fMRI of the Brain, Oxford, UK). Bilateral cortical ROIs were created in MNI space using the Brainnetome atlas [69] with probability maps thresholded at 35%. The M1 ROI included areas A4ul (upper limb) and A4hf (hand function) while the PMC comprised areas A6cdl (dorsal PMC) and A6cvl (ventral PMC). The PER included A35_36c (caudal area) and A35_36r (rostral area) as in [70] and PHC comprised both TH (medial posterior PHC) and TL (lateral posterior PHC) areas [69]. The resulting ROI masks were mapped back to individual native space using the individual’s inverse deformation field output from the segmentation of the anatomical image. All ROIs were registered to the functional volumes and masked to exclude voxels outside of the brain. Voxels showing less than 10% grey matter probability were also excluded from the ROIs. The average number of voxels within each ROI is reported in S6 Table.

##### Representational similarity analyses (RSA).

To assess coding of temporal order and item information during sequence learning within and across memory domains, we used multivoxel pattern analyses of task-related fMRI data. In particular, we used representational similarity analyses (RSA) which are based on the assumption that if a particular type of information is processed by a particular brain region, then it will be represented in the pattern of activation across voxels in that brain region [22]. Such analyses therefore predict higher correlations in voxel activity between pairs of trials sharing this information as compared to pairs of trials not sharing this information. Using RSA on the sequence condition data, we first assessed the extent to which our ROIs represent information about memory items in their learned temporal position in a sequence (1) **within the motor memory domain**, i.e., representation of fingers in their learned position in the motor sequence referred to as *finger-position coding*, (2) **within the declarative memory domain**, i.e., representation of objects in their learned position in the object sequence referred to as *object-position coding*, and (3) **across memory domains**, i.e., representation of items (irrespective of the memory domain) in their learned position in the sequence referred to as *item-position coding*. Next, using the random condition data, we performed control analyses to assess to what extent the different ROIs also represented information about finger/object and position. Specifically, we tested whether the ROIs carry information about (1) fingers irrespective of their temporal position in the stream of movement (i.e., finger coding), (2) objects irrespective of their temporal position in the stream of objects (object coding); and (3) temporal positions irrespective of finger/object information (i.e., position coding).

To perform the RSA, 3 separate GLMs were fitted to the 8 runs of preprocessed functional data of each participant. The first GLM (i.e., position GLM) was constructed to model neural responses evoked for each temporal position and include one regressor for each position (8 temporal positions in the stream repeated 5 times) for each of the 3 conditions (motor sequence, object sequence and random) in each run (8 positions × 3 conditions = 24 regressors per run, representing positions in motor sequence, positions in object sequence and positions in random condition; 8 runs, i.e., 192 regressors in total). Within each task condition, each position was presented 5 times per run, which resulted in 5 events for each of the 192 regressors. Similarly, the second GLM (i.e., key GLM) was constructed to model neural responses evoked by individual key presses and include one regressor for each finger/key for each condition in each run (8 × 3 regressors = 24 regressors per run, representing keys in motor sequence, keys in object sequence and keys in random condition with 5 events for each regressor; 8 runs, i.e., 192 regressors in total). Finally, a third GLM (i.e., object GLM) was constructed to model neural responses evoked by individual objects and include one regressor for each object for each condition in each run (8 × 3 regressors = 24 regressors per run, representing objects in motor sequence, objects in object sequence and objects in random condition with 5 events for each regressor; 8 runs, i.e., 192 regressors in total). Since the finger-position and object-position associations are fixed in the object and motor sequence conditions, the same events were modelled in the 3 different GLMs for the sequence conditions. As there is no such association in the random condition, events were reclassified according to (i) temporal position (irrespective of key or object; position GLM), (ii) key pressed (irrespective of temporal position and object; key GLM) or (iii) object (irrespective of key and temporal position; object GLM). For all GLMs, neural responses to events of interest were modelled with delta functions (0 ms duration) time locked to cue onsets. Movement parameters and responses during rest periods were entered as nuisance regressors. High-pass filtering with a cutoff of 128 s was used to remove low-frequency drifts from the time series. An autoregressive (order 1) plus white noise model and a restricted maximum likelihood (ReML) algorithm was used to estimate serial correlations in the fMRI signal. The GLMs resulted in separate maps of *t*-values for each regressor in each run for each ROI. For each voxel within each ROI, the *t*-values were normalized by subtracting the mean *t*-value across all regressors within each run separately.

To estimate neural similarity matrices for each ROI, the full dataset (8 runs) was randomly divided into two independent subsets of 4 runs and *t*-values were averaged within each set to give 2 sets of *t*-values for each regressor for each voxel. For a given condition within one of the GLMs, the *t*-values for all the voxels within an ROI from one set was correlated with the second set for all combinations of the 8 regressors for that condition (e.g., position 1–8). This resulted in an 8 × 8 matrix of Pearson correlation coefficients. This procedure was repeated 140 times (i.e., 2 conditions × 70 possible combinations when choosing 4 numbers out of 8), each time averaging over different subsets of runs. The correlation coefficients were fisher-transformed and then averaged across the 140 iterations resulting in an 8 × 8 correlation matrix for each participant, ROI, and research question of interest. A total of 7 matrices were constructed to address the different research questions, i.e., which ROIs code for (1) finger-position coding (within motor sequence condition), (2) object-position coding (within object sequence condition), (3) item-position coding (between motor and object sequence conditions), (4) temporal position (within random condition), (5) key (within random condition), and (6) object (within random condition). Note that an additional control matrix was created to test for (7) item coding (within random condition) to control for the potential contribution of item similarity between memory domains (e.g., any unexpected similarity – during random practice – between, e.g., key 2 and elephant items that are presented in the same temporal position in the sequence condition) to the results observed in the item-position matrix (see Figs 3–5).

We first examined ***selective coding*** in our different ROIs, i.e., whether voxel activity patterns were more similar during the repetition of trials sharing the same information as compared to pairs of trials not sharing this information. Since the diagonal of the correlational matrix represents a correlation between the same information (e.g., key 4 versus key 4), if a brain region codes for this type of information, then we would expect a high similarity index when compared to correlations between information that is different (off-diagonal, e.g., key 4 versus key 1). To investigate whether a given brain region coded for a particular type of information, for each ROI and each type of matrix, a one-tailed paired sample *t* test was therefore performed across participants to compare diagonal versus non-diagonal mean similarity.

Next, when the ROIs presented evidence of coding for multiple representations (e.g., item-position in the sequence condition and item coding in the random condition), we assessed the contribution of the different overlapping representations derived from random practice to the coding results observed in the sequence condition using forward stepwise linear regression analyses. Specifically, the goal of these analyses was to identify whether item (finger, object, item) and/or position coding (as assessed during random practice) can explain pattern similarity effects in the sequence conditions. To do so, we ran separate models using delta similarity (i.e., mean pattern similarity diagonal minus off-diagonal) for finger-position/object-position/item-position coding as a dependent variable and delta similarity for finger/object/item and position matrices as predictors. When two predictors were included in the model, an *F*-test was used to assess whether the change in explained variance (i.e., *R*^2^ change; Δ*R*^2^) from the prior step was significant. Full models including the two predictors within each memory domain and across memory domains can be found for each ROI in S2–S4 Tables.

For all the statistical analyses described above, we applied Bonferroni correction to correct for the 5 ROIs. Note that for motor and object sequence learning, the results reported in the main text only included the 3 memory-domain specific ROIs (i.e., 3 motor ROIs: M1, PMC and HC for motor sequence and 3 object ROIs: PER, PHC and HC for object sequence) but still corrected for 5 ROIs. The supplemental information (S1–S2 Figs) provides results for all ROIs within each memory domain (i.e., motor sequence learning RSA on object ROIs and object sequence learning RSA on motor ROIs) for completeness.

##### Supplementary control analyses and experiments.

*Control behavioral experiment assessing the explicit knowledge of the series of objects and movements:* To better characterize sequence learning in this new paradigm, we collected data from an independent sample of participants with a design that closely mirrored the one used in the current study but with the addition of generation tasks similar as in earlier research [15,71]. Specifically, the object generation/recall tasks aimed at reconstructing the temporal order of the object items and the motor generation task tested for any explicit knowledge of the learned sequences of movements. The detailed methods and results corresponding to this control experiment are presented in the supplemental information (Section C in S1 Text and S4 Fig). The results of this separate control experiment show that participants were able to recall/generate the series of both objects and movements with high accuracy (>89%). These results complement the response-based data presented in the manuscript and indicate that participants developed explicit knowledge of the series of objects and movements with this paradigm.

*Performance speed and similarity patterns*: As performance differed between task conditions during initial training (see “Results” section), we examined whether the level of performance reached at the end of the training session on day 1 was correlated with neural pattern similarity examined the next day (note though that performance did not differ between conditions during the scanning session). These control analyses are presented in Section D in S1 Text and indicate that the level of performance reached on day 1 is unlikely to influence the pattern of brain results observed the next day.

*Effect of lag on pattern similarity:* To assess whether pattern similarity changed as a function of the lag between items in the learned sequences (e.g., one could expect a gradient of temporal representations such that items that are one position apart are more similar than items two or more positions apart in the sequence [15]), we performed fine-grained analyses of the patterns on the off-diagonal cells in the across sequence matrices for each ROI. To mirror the analyses reported in the main text, we also tested (1) whether there were lag-dependent effects in the random position and/or random item matrices, and (2) whether these off-diagonal patterns were predictive of the off-diagonal patterns in the across sequence matrix. The corresponding methods and results are reported in the supplementary material (see Section E in S1 Text and S5 Fig). In summary, these analyses show that although there was no progressive decrease in similarity with distance between learned items in any of the ROIs, consecutive items showed greater dissimilarity which is partly in line with our earlier observations [16]. Importantly, the results of the off-diagonal pattern analyses closely mirrored the diagonal results as they show that off-diagonal patterns across memory domains could be attributed to position coding in M1, PMC and PHC but not in HC and PER. These results therefore suggest that off-diagonal patterns in the HC and PER might reflect information about temporal order in a sequence across memory domains.

*Univariate analyses of the fMRI data*: As Pearson correlation approaches are known to be an unreliable measure of pattern similarity when there are differences in mean univariate activation among conditions [72], we performed control univariate analyses to confirm that this was not an issue in our study. To do so, SPM12 was used to fit a GLM to each individual’s data with the neural responses in the motor (SQ MOT), object (SQ OBJ) and random (RD) task conditions modelled as three separate regressors. Single subject contrast maps obtained from this first-level analysis and testing for the main effect of task condition were then entered into a second-level random effects analysis. To test for differences in activation levels between conditions in our ROIs, the resulting t statistics maps [SPM(T)] were masked inclusively with a large mask that included all five ROIs. No suprathreshold clusters were found in any of the contrasts (i.e., SQ MOT > SQ OBJ, SQ OBJ > SQ MOT, SQ OBJ > RD, RD > SQ OBJ, SQ MOT > RD or RD > SQ MOT) in any of our ROIs (FWE corrected for multiple comparisons across the number of voxels in the mask). Accordingly, there are no significant differences in activity levels among conditions in our ROIs and thus Pearson correlations are considered a suitable approach to perform multivoxel pattern analyses in the current dataset.

*Surrogate analyses*: Surrogate control matrices were computed to provide an estimation of random/baseline data. The corresponding methods are described in Section F in S1 Text. Briefly, for each individual, these surrogate neural similarity matrices were created by randomly shuffling the labels of the fingers within the SQ MOT and RD KEY matrices, objects within the SQ OBJ and RD OBJ matrices, items within the SQ ACROSS and RD ITEM matrices and positions within the RD POS matrix (1,000 permutations within each matrix). In a first step, we replicated the diagonal versus off diagonal statistical analyses presented in the main text to assess a baseline effect on surrogate matrices (obtained by averaging over all 1,000 permutations, see S6 Fig). Results show that there were no significant differences in mean pattern similarity between diagonal and off-diagonal cells of the surrogate matrices for any of the conditions in any of the ROIs, see S7 Fig). Next, we tested whether the pattern similarity effects observed in the current study exceeded what would be expected based on random noise (modelled in the surrogate matrices). To do so, matrices obtained in the main analyses were recalculated for each individual by subtracting their corresponding surrogate matrix (obtained by averaging over all 1,000 permutations). The results are identical to those derived from the original analyses (see Section F in S1 Text).

*Cross-validation*: The reliability of the similarity measures (Pearson correlations) reported in this manuscript was assessed using an approach of split-half reliability estimates recommended by [72]. The detailed methods and corresponding results are presented in Section G in S1 Text and S8 Fig. Briefly, the RSA split-half reliability measures were in the range of what was previously observed for such representational similarity analyses [72] and the results showed moderate to good reliability for the matrices corresponding to the information coded in the specific brain regions.

*Brain patterns related to the visual processing of the central object*: As we did not observe object coding in any of our object ROIs, we performed supplemental analyses to test whether there was successful visual processing of the central object image. Specifically, we tested whether the well-known object-category representation described in the ventral occipito-temporal cortex VOTC (e.g., [32,33]) could be observed in our dataset. To do so, we computed the object representational similarity matrix from the random data (8 × 8 ***RD OBJ*** matrix) in a VOTC ROI that was defined using the Brainnetome atlas [69] by combining fusiform (37mv, 37lv, A20rv) and infero-temporal areas (A20iv, A37elv, A20r, A20il, A37vl, A20cl, A20cv). As for the other object ROIs, object coding was assessed with the comparison between the mean similarity averaged along the diagonal (i.e., same object) and mean similarity averaged from all off-diagonal cells (i.e., different objects). We also assessed object-category coding and compared mean similarities between objects belonging to the same categories (e.g., similarity between the two fruits; pineapple and banana) with the mean similarity between objects belonging to different categories (e.g., similarity between banana and plane). We also compared similarity values averaged across different object categories to similarity within each object category. These results presented in S3 Fig show that we were able to observe the well-known object-category representation in the VOTC (e.g., [32,33]) which suggests successful visual processing of the central image in the current study.

*Analyses of eye movements:* Gaze coordinates were extracted from fMRI data using DeepMReye toolbox [73] which uses a convolutional neural network trained to decode gaze location directly from the MR signal of the eyeballs. Pre-trained weights, originally trained on datasets that included simultaneous BOLD fMRI and eye-tracking data were applied to our data to extract gaze coordinates from each volume across all runs and participants. Gaze coordinates were used to assess: (1) *the mean distance travelled by the eyes for each task condition* in order to control that the quantity of eye movements was similar between tasks and (2) *gaze patterns for each condition* in order to better characterize the visual strategy used during sequence learning. The methods and results corresponding to these control analyses are presented in the supplemental information (Section H in S1 Text and S9–S10 Figs). Briefly, the results of these control analyses suggest that the quantity of eye movements was similar between task conditions and therefore unlikely introduced a confound in the data. Gaze analyses suggest that participants used a similar visual search strategy for the two sequence conditions. They also show decreased central fixation time during object sequence learning – as compared to the random task – which suggests that the knowledge of the series of objects resulted in a decreased need to fixate the central cue to complete the task.

## Supporting information

S1 Data Source data for the main figures. The source data corresponding to the figures presented in this manuscript are available as supporting information under the file name “S1_Data.xlsx.” The raw behavioral and demographic data and analysis codes are publicly available at [https://doi.org/10.5281/zenodo.15642711].
(XLSX)

S2 Data Source data for the supporting figures. The source data corresponding to the figures presented in the supporting information are available as supporting information under the file name “S2_Data.xlsx.”
(XLSX)

S1 Fig Similarity matrices. Group average neural similarity matrices for all the ROIs. Pattern similarity was computed across repetitions of the motor sequence to assess finger-position coding in the sequence condition (SQ MOT matrix), across repetitions of the object sequence to assess object-position coding in the sequence condition (SQ OBJ matrix), between pairs of the individual objects from the object sequence condition and individual fingers from the motor sequence condition to assess item-position coding across sequences from different domains (SQ ACROSS matrix), as well as across repetitions of the random patterns to quantify finger/key (RD KEY matrix), position (RD POS matrix), object (RD OBJ matrix) and item (RD ITEM) coding in the random condition. Color bar represents mean similarity (r). In the POS rows/columns, numbers represent the temporal position in a sequence. In the KEY rows/columns, numbers represent fingers. In the OBJ rows/columns, the first 2 letters of each object are presented. (X) represents a random position or key/object in the item and position matrices, respectively. The SQ matrices represents the sequences performed by an exemplary individual (motor sequence: key 4 in position 1, key 7 in position 2, etc. and object sequence: pineapple in position 1, plane in position 2, etc.). The source data corresponding to this figure are included in S2 Data, “S1 Fig” sheet.
(TIF)

S2 Fig Mean pattern similarity for diagonal (blue) and off-diagonal (red) cells as a function of matrices and ROIs. Results indicate that all ROIs (primary motor cortex (M1), premotor cortex (PMC), parahippocampus (PHC), perirhinal (PER), hippocampus (HC)) show evidence of finger-position, object-position and item-position coding in the sequence conditions. M1, PMC and PHC show evidence of finger and position coding during random practice whereas HC and PER do not. None of the ROIs showed evidence of object or item coding. Asterisks indicate significant differences between diagonal and off-diagonal (one sided paired sample *t* test; Bonferroni corrected *p_*corr*_ < .05 and ** *p*_*corr*_ < .005). Colored circles represent individual data, jittered in arbitrary distances on the x-axis to increase perceptibility. Horizontal lines represent means and white circles represent medians. The shape of the violin [23] depicts the kernel density estimate of the data. Note that as in earlier research [16], *Y* axis scales are different between ROIs to accommodate for differences in signal-to-noise ratio (and therefore in effect sizes) between ROIs. The source data corresponding to this figure are included in S2 Data, “S2 Fig” sheet.
(TIF)

S3 Fig Brain patterns related to the visual processing of the central object. **(A)** Group average neural similarity matrices for objects using random data in the perirhinal cortex (PER) and the ventro-occipo-temporal cortex (VOTC) to assess object/object category coding. Colored squares along the diagonal represent correlations between object within the same category, i.e., fruit (yellow outline), animal (purple outline), vehicle (green outline) and tool (blue outline) categories. In the rows/columns, the first 2 letters of each object are presented. **(B)** Left panel. Mean pattern similarity for diagonal (correlation between repetitions of the same object) and off-diagonal (correlation between repetition of different objects) cells. In line with the results presented in the main text, these analyses indicate that neither PER nor VOTC showed evidence of object coding. Right panel. Mean pattern similarity between different object categories (diffcat, see dark blue frame in matrices presented in panel **A**), between same object categories (samecat, i.e., across all cells highlighted with the four colored frames along the diagonal in matrices presented in panel **A**) and within each of the object categories (fruit, animal, vehicle, tool). Results showed a main effect of category (same versus different) in the VOTC (*t*(29) = 2.78, *d* = 0.51, *p* = 0.01) which confirms the well-known object category coding in this region (see “Discussion” in main text). This effect was particularly pronounced for the animal (animal category versus different category, *t*(29) = 4.25, *d* = 0.78, *p* < 0.002) and tool (tool category versus different category, *t*(29) = 2.25, *d* = 0.41, *p* = 0.04) categories. No such effects were observed in the PER. Asterisks indicate significant differences (one sided paired sample *t* test; Bonferroni corrected *p_*r*_ < .05 and **p_*r*_ < .005). Colored circles represent individual data, jittered in arbitrary distances on the *x*-axis to increase perceptibility. Horizontal lines represent means and white circles represent medians. The shape of the violin [23] depicts the kernel density estimate of the data. The source data corresponding to this figure are included in S2 Data, “S3 Fig” sheet.
(TIF)

S4 Fig Behavioral results from separate control behavioral experiment. Response time in seconds (top panel) and accuracy (% correct response, bottom panel) across blocks and sessions for all task conditions. Dark blue: object sequence (SQ OBJ), light blue: motor sequence (SQ MOT), pink: random blocks (RD) (darker shade corresponds to random practice during the object session). Shaded error bars = SEM. (*n* = 24). The source data corresponding to this figure are included in S2 Data, “S4 Fig” sheet.
(TIF)

S5 Fig Pattern similarity as a function of lag. **(A)** Pattern similarity extracted for the across sequences (top), random position (middle) and random item (bottom) matrix as a function of lag in the learned sequences for M1, PMC, PER, PHC and HC. For each ROI, the leftmost bar illustrates mean pattern similarity across repetitions of the same item (key/object) in the same position (“lag 0”). The other bars illustrate pattern similarity across repetitions of items (key/objects) separated by one (“lag 1”), two (“lag 2”), three (“lag 3”) or more (“lag 4”, “lag 5”, “lag 6”, “lag 7”) positions in the learned sequence. **SQ ACROSS matrix**: Results indicate a main effect of lag in all ROIs (M1: *F*_(7,203)_ = 60.22, *ɳ*_*p*_^2^ = .68, *p*_*corr*_ < .005; PMC: *F*_(7,203)_ = 82.54, *ɳ*_*p*_^2^ = .74, *p*_*corr*_ < .005; PHC: *F*_(7,203)_ = 146, *ɳ*_*p*_^2^ = .83, *p*_*corr*_ < .005; PER: *F*_(7,203)_ = 140.2, *ɳ*_*p*_^2^ = .83, *p*_*corr*_ < .005; HC: *F*_(7,203)_ = 161.26, *ɳ*_*p*_^2^ = .85, *p*_*corr*_ < .005). Follow-up analyses indicate that lag 0 was greater than all other lags (all *p*_*corr*_ < .05) except for lag 0 versus lag 6 in M1, PHC, PER and HC (all *p*_*corr*_ = 1) and also between lag 0 versus lag 4 in PER, *p*_*corr*_ = .1 and HC, *p*_*corr*_ = 1). Lag 7 showed lower similarity than all other lags (all *p*_*corr*_ < .05) in all ROIs. In M1 and PMC, a decrease in similarity was observed in odd lags 1, 3 and/or 5 compared to other lags (M1: lags 1, 5 < 2, 6, all *p*_*corr*_ < .05; PMC: lag 5 < 2, 3, 4 and lags 1, 3 < 2, all *p*_*corr*_ < .05). This effect was less pronounced in the PHC, PER and HC, where a decrease in similarity was only observed in lag 5 compared to other lags (lag 5 < 2–4, all *p*_*corr*_ < .05 and lag 5 < 1, in HC only, *p*_*corr*_ < .005). In the HC, an increase in similarity was also observed in even lags 4 and 6 compared to lags 1, 3 and/or 5 (all *p*_*corr*_ < .005). **RD POS matrix**: Results indicate a main effect of lag in all ROIs except HC and PER (M1: *F*_(7,203)_ = 19.1, *ɳ*_*p*_^2^ = .4, *p*_*corr*_ < .005; PMC: *F*_(7,203)_ = 30.3, *ɳ*_*p*_^2^ = .5, *p*_*corr*_ < .005; PHC: *F*_(7,203)_ = 4.61, *ɳ*_*p*_^2^ = .14, *p*_*corr*_ < .005; HC: *F*_(7,203)_ = 1.9, *ɳ*_*p*_^2^ = .06, *p*_*corr*_ = .34; PER: *F*_(7,203)_ = 2.31, *ɳ*_*p*_^2^ = .07, *p*_*corr*_ = .14). Planned pairwise comparisons indicate that in M1 and PMC, similarity values of the diagonal (i.e., lag 0, same position) were significantly larger than all other lags (i.e., lag 0 > all other lags, all *p*_*corr*_ < .05), while in the PHC, the diagonal was only significantly larger than lag 2 (*p*_*corr*_ < .05). M1 and PMC showed a large reduction in pattern similarity on lag 7 as compared to all other lags (all *p*_*corr*_ < .05), except for lag 6 in M1 (*p*_*corr*_ = .13). **RD ITEM matrix**: Results did not show any lag-dependent effects in the random item matrix for any of the ROIs (M1: *F*_(7,203)_ = 1.21, *ɳ*_*p*_^2^ = .04, *p*_*corr*_ = 1; PMC: *F*_(7,203)_ = 1.39, *ɳ*_*p*_^2^ = .05, *p*_*corr*_ = 1; PHC: *F*_(7,203)_=.97, *ɳ*_*p*_^2^ = .03, *p*_*corr*_ = 1; PER: *F*_(7,203)_ = 1.1, *ɳ*_*p*_^2^ = .04, *p*_*corr*_ = 1; HC: *F*_(7,203)_ = 0.8, *ɳ*_*p*_^2^ = .03, *p*_*corr*_ = 1). Colored circles represent individual data, jittered in arbitrary distances on the *x*-axis to increase perceptibility and white circles represent medians. The shape of the violin depicts the kernel density estimate of the data. **(B)** Results of correlations between off-diagonal cells from the across sequence matrix and (1) the random position (Pos) and the (2) random item (Item) matrices (using non-diagonal cells from lags 1–7 averaged across upper and lower triangles) for M1, PMC, PER, PHC and HC. For each matrix, one sample *t*-tests were used to test whether the average correlation was significantly different from 0. Results of two-tailed one sample *t*-tests were corrected for multiple comparisons, considering the number of ROIs. **p < .005, *p < .05. Error bars denote ±1 SEM. The source data are included in S2 Data, “S5 Fig” sheet.
(TIF)

S6 Fig Group average surrogate neural similarity matrices. Surrogate matrices by condition (i.e., SQ MOT, SQ OBJ, SQ ACROSS, RD KEY, RD POS, RD OBJ and RD ITEM) for all ROIs. Color bars represent mean similarity (*r*). The source data corresponding to this figure are included in S2 Data, “S6 Fig” sheet.
(TIF)

S7 Fig Mean pattern similarity for diagonal and off-diagonal cells in the surrogate matrices. For all ROIs and all condition there were no significant differences in mean pattern similarity between diagonal and off-diagonal cells. Colored circles represent individual data, jittered in arbitrary distances on the *x*-axis to increase perceptibility and white circles represent medians. The shape of the violin [23] depicts the kernel density estimate of the data. The source data corresponding to this figure are included in S2 Data, “S7 Fig” sheet.
(TIF)

S8 Fig Group reliability measures. Reliability measures for each condition (i.e., SQ MOT, SQ OBJ, SQ ACROSS, RD KEY, RD POS, RD OBJ and RD ITEM) for each ROI. The measure of reliability was calculated as the similarity between the matrices resulting from a half split cross validation analysis. Higher *r* values represent higher reliability. Error bars denote ±1 SEM. Arrows indicates the condition that is compared to the other conditions. *p_*corr*_ < .05 and **p_*corr*_ < .01. The source data corresponding to this figure are included in S2 Data, “S8 Fig” sheet.
(TIF)

S9 Fig Group mean distance travelled by eyes. **(A)** Mean distance (in degrees of visual angle) travelled by the eyes across runs of task (motor, object and random) and runs of rest. **(B)** Mean distance travelled by the eyes across runs per condition (motor, object and random). Shaded regions denote ±1 SEM. The source data corresponding to this figure are included in S2 Data, “S9 Fig” sheet.
(TIF)

S10 Fig Gaze heatmaps. **(A)** Gaze heatmaps for the motor sequence, object sequence, random and rest conditions averaged across the 8 MRI runs. Brighter values represent longer fixation durations. **(B)** Differential heat maps between the motor versus object sequence conditions, the motor sequence versus random conditions, the object sequence versus random conditions, and between the task (motor, object and random) versus the rest condition. Clusters representing significant differences in gaze patterns between conditions are highlighted in darker color in the object versus random and task versus rest comparisons. Note that as we did not perform any calibration and pre-trained data was used to extract gaze coordinates, we cannot conclude on the specific gaze location in the different conditions. Accordingly, we only reflect as to whether fixation was more central or lateral (e.g., task versus rest contrast). The source data corresponding to this figure are included in S2 Data, “S10 Fig” sheet.
(TIF)

S1 Table Control analyses on 6 × 6 matrices after removing boundary positions. One-tailed one sample-test results for the comparison of delta similarity (mean diagonal cells versus mean off-diagonal cells) against zero for position matrices after edge removal (6 × 6 matrices). After removing boundary positions, position coding only remained significant in the premotor cortex while such coding was no longer significant in all other ROIs. *P* values are Bonferroni-corrected. The source data used to perform the statistical tests reported in this table are included in S2 Data, “S1 Table” sheet.
(TIF)

S2 Table Stepwise linear regression results for motor sequence learning for all ROIs. Dependent variable: delta similarity (i.e., difference between mean diagonal and mean off-diagonal cells) in the motor sequence matrix. Predictors: delta similarity in the random key and/or position matrix. An *F*-test was used to assess whether the change in explained variance (Δ*R*^2^) from the prior step is significant. Step 1, *df*1 = 1, *df*2 = 28; Step 2, *df*1 = 2, *df*2 = 27. *P*-values are Bonferroni corrected. The source data used to perform the statistical tests reported in this table are included in S2 Data, “S2 Fig” sheet.
(TIF)

S3 Table Stepwise linear regression results for object sequence learning for all ROIs. Dependent variable: delta similarity (i.e., difference between mean diagonal and mean off-diagonal cells) in the object sequence matrix. Predictors: delta similarity in the random object and/or position matrix. An *F*-test was used to assess whether the change in explained variance (Δ*R*^2^) from the prior step is significant. Step 1, *df*1 = 1, *df*2 = 28; Step 2, *df*1 = 2, *df*2 = 27. *P*-values are Bonferroni corrected. The source data used to perform the statistical tests reported in this table are included in S2 Data, “S2 Fig” sheet.
(TIF)

S4 Table Stepwise linear regression results for sequence learning across domains for all ROIs. Dependent variable: delta similarity (i.e., difference between mean diagonal and mean off-diagonal cells) in the item-position matrix. Predictors: delta similarity in the random item and/or position matrix. An *F*-test was used to assess whether the change in explained variance (Δ*R*^2^) from the prior step is significant. Step 1, *df*1 = 1, *df*2 = 28; Step 2, *df*1 = 2, *df*2 = 27. *P*-values are Bonferroni corrected. The source data used to perform the statistical tests reported in this table are included in S2 Data, “S2 Fig” sheet.
(TIF)

S5 Table Participant demographics, sleep and vigilance characteristics. *Notes.* Values are means and standard deviations (unless noted otherwise). Cutoff values for exclusion in parentheses. PSQI = Pittsburgh Sleep Quality Index. CRQ = Circadian Rhythm Questionnaire. ^a^Determined in combination between sleep diary and wrist actigraphy recordings on night 4 (between the two experimental sessions). ^b^1 = very badly, 6 very well. ^c^Median of reaction times. ^d^1 = more alert, 7 = less alert. The source data corresponding to this table are included in S2 Data, “S5 Table” sheet.
(TIF)

S6 Table ROI size (number of voxels). The source data corresponding to this table are included in S2 Data, “S6 Table” sheet.
(TIF)

S7 Table Participant demographics, sleep and vigilance characteristics for Control behavioral experiment. *Notes.* Values are means and standard deviations (unless noted otherwise). Cutoff values for exclusion in parentheses. PSQI = Pittsburgh Sleep Quality Index. CRQ = Circadian Rhythm Questionnaire. ^a^Determined in combination between sleep diary and wrist actigraphy recordings on night 4 (between the two experimental sessions). ^b^1 = very badly, 6 very well. ^c^Median of reaction times. ^d^1 = more alert, 7 = less alert. The source data corresponding to this table are included in S2 Data, “S7 Table” sheet.
(TIF)

S1 Text Supplementary analyses. (DOCX)

## Abbreviations

fMRI: functional magnetic resonance imaging
MVPA: multivariate pattern analyses
PVT: psychomotor vigilance test
ROI: region of interest
RSA: representational similarity analysis
SRTT: serial reaction time task
