# Supplementary figures and images for: The human medial temporal lobe represents memory items in their ordinal position in both declarative and motor memory domains
Source: PLoS Biol. 2025 Jul 7;23(7):e3003267. doi: 10.1371/journal.pbio.3003267 (PMC12258568; doi:10.1371/journal.pbio.3003267)

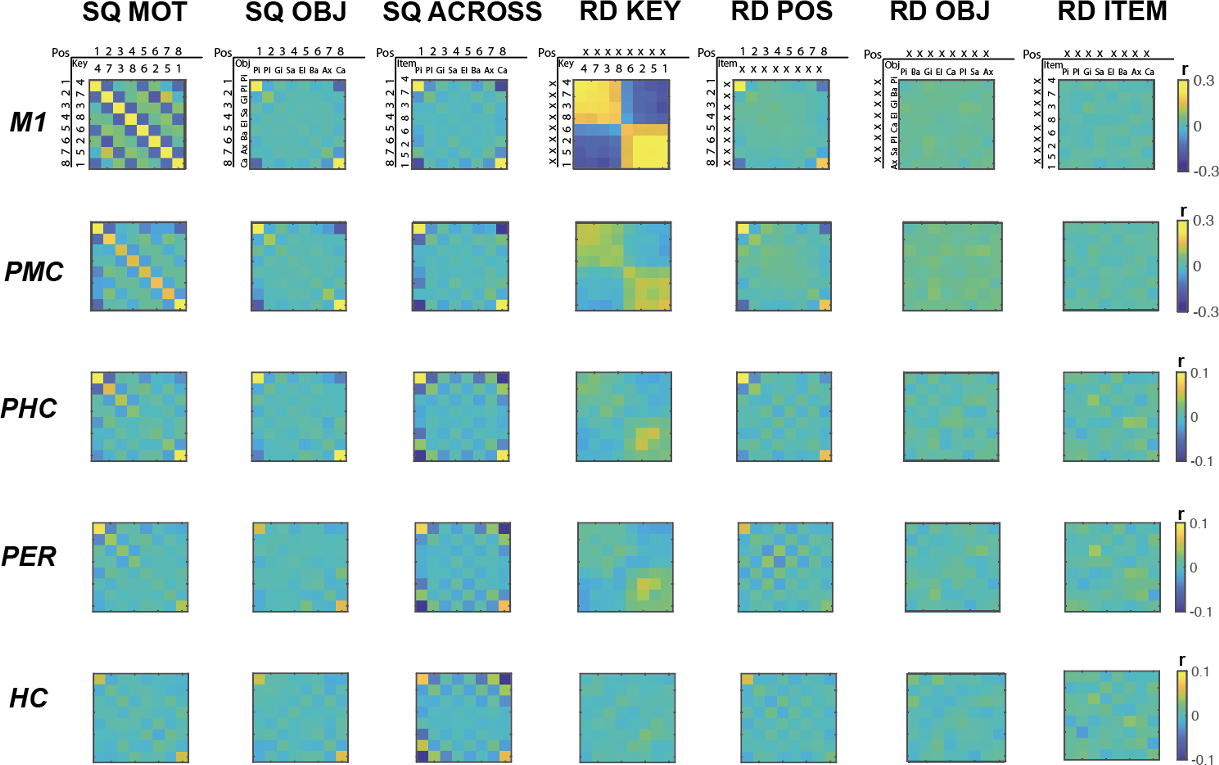

Supplement: S1 Fig — Similarity matrices. Group average neural similarity matrices for all the ROIs. Pattern similarity was computed across repetitions of the motor sequence to assess finger-position coding in the sequence condition (SQ MOT matrix), across repetitions of the object sequence to assess object-position coding in the sequence condition (SQ OBJ matrix), between pairs of the individual objects from the object sequence condition and individual fingers from the motor sequence condition to assess item-position coding across sequences from different domains (SQ ACROSS matrix), as well as across repetitions of the random patterns to quantify finger/key (RD KEY matrix), position (RD POS matrix), object (RD OBJ matrix) and item (RD ITEM) coding in the random condition. Color bar represents mean similarity (r). In the POS rows/columns, numbers represent the temporal position in a sequence. In the KEY rows/columns, numbers represent fingers. In the OBJ rows/columns, the first 2 letters of each object are presented. (X) represents a random position or key/object in the item and position matrices, respectively. The SQ matrices represents the sequences performed by an exemplary individual (motor sequence: key 4 in position 1, key 7 in position 2, etc. and object sequence: pineapple in position 1, plane in position 2, etc.). The source data corresponding to this figure are included in S2 Data, “S1 Fig” sheet. (TIF) [file pbio.3003267.s003.tif]

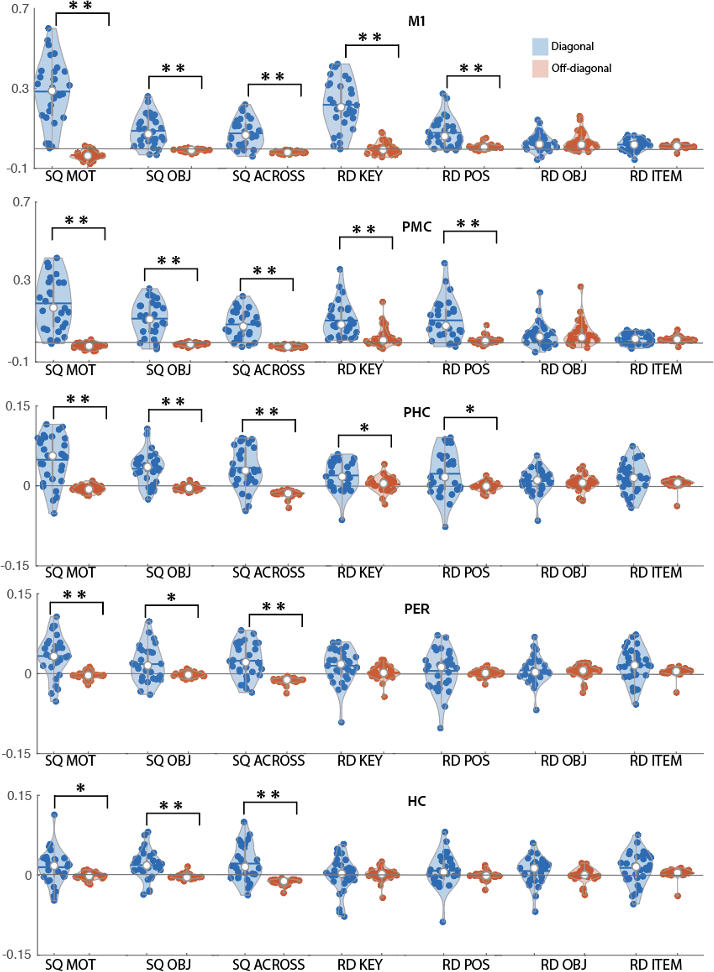

Supplement: S2 Fig — Mean pattern similarity for diagonal (blue) and off-diagonal (red) cells as a function of matrices and ROIs. Results indicate that all ROIs (primary motor cortex (M1), premotor cortex (PMC), parahippocampus (PHC), perirhinal (PER), hippocampus (HC)) show evidence of finger-position, object-position and item-position coding in the sequence conditions. M1, PMC and PHC show evidence of finger and position coding during random practice whereas HC and PER do not. None of the ROIs showed evidence of object or item coding. Asterisks indicate significant differences between diagonal and off-diagonal (one sided paired sample t test; Bonferroni corrected *pcorr < .05 and ** pcorr < .005). Colored circles represent individual data, jittered in arbitrary distances on the x-axis to increase perceptibility. Horizontal lines represent means and white circles represent medians. The shape of the violin [23] depicts the kernel density estimate of the data. Note that as in earlier research [16], Y axis scales are different between ROIs to accommodate for differences in signal-to-noise ratio (and therefore in effect sizes) between ROIs. The source data corresponding to this figure are included in S2 Data, “S2 Fig” sheet. (TIF) [file pbio.3003267.s004.tif]

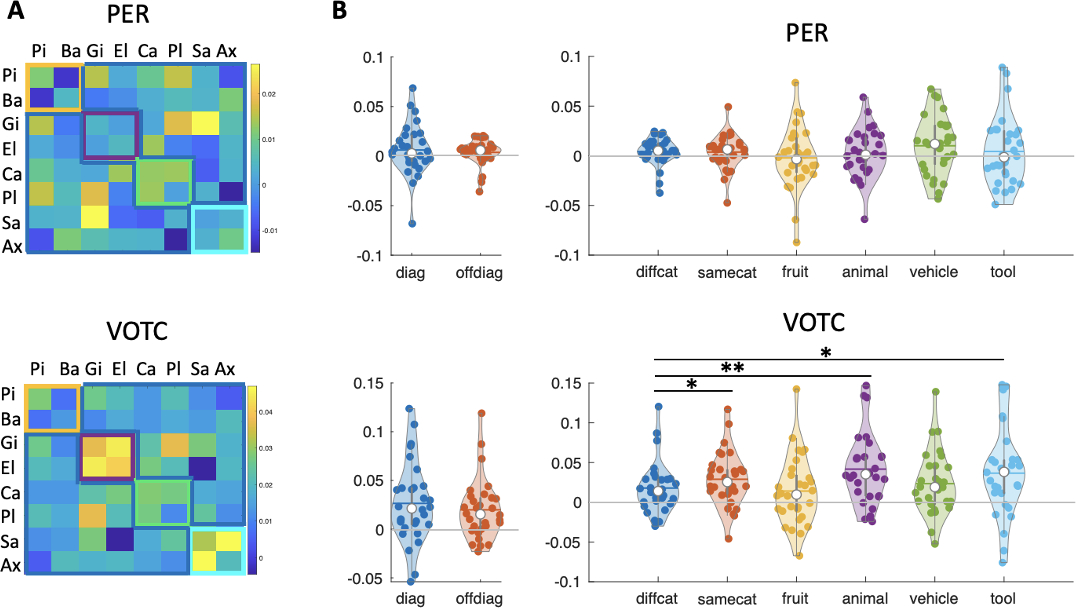

Supplement: S3 Fig — Brain patterns related to the visual processing of the central object. (A) Group average neural similarity matrices for objects using random data in the perirhinal cortex (PER) and the ventro-occipo-temporal cortex (VOTC) to assess object/object category coding. Colored squares along the diagonal represent correlations between object within the same category, i.e., fruit (yellow outline), animal (purple outline), vehicle (green outline) and tool (blue outline) categories. In the rows/columns, the first 2 letters of each object are presented. (B) Left panel. Mean pattern similarity for diagonal (correlation between repetitions of the same object) and off-diagonal (correlation between repetition of different objects) cells. In line with the results presented in the main text, these analyses indicate that neither PER nor VOTC showed evidence of object coding. Right panel. Mean pattern similarity between different object categories (diffcat, see dark blue frame in matrices presented in panel A), between same object categories (samecat, i.e., across all cells highlighted with the four colored frames along the diagonal in matrices presented in panel A) and within each of the object categories (fruit, animal, vehicle, tool). Results showed a main effect of category (same versus different) in the VOTC (t(29) = 2.78, d = 0.51, p = 0.01) which confirms the well-known object category coding in this region (see “Discussion” in main text). This effect was particularly pronounced for the animal (animal category versus different category, t(29) = 4.25, d = 0.78, p < 0.002) and tool (tool category versus different category, t(29) = 2.25, d = 0.41, p = 0.04) categories. No such effects were observed in the PER. Asterisks indicate significant differences (one sided paired sample t test; Bonferroni corrected *pr < .05 and **pr < .005). Colored circles represent individual data, jittered in arbitrary distances on the x-axis to increase perceptibility. Horizontal lines represent means a [file pbio.3003267.s005.tif]

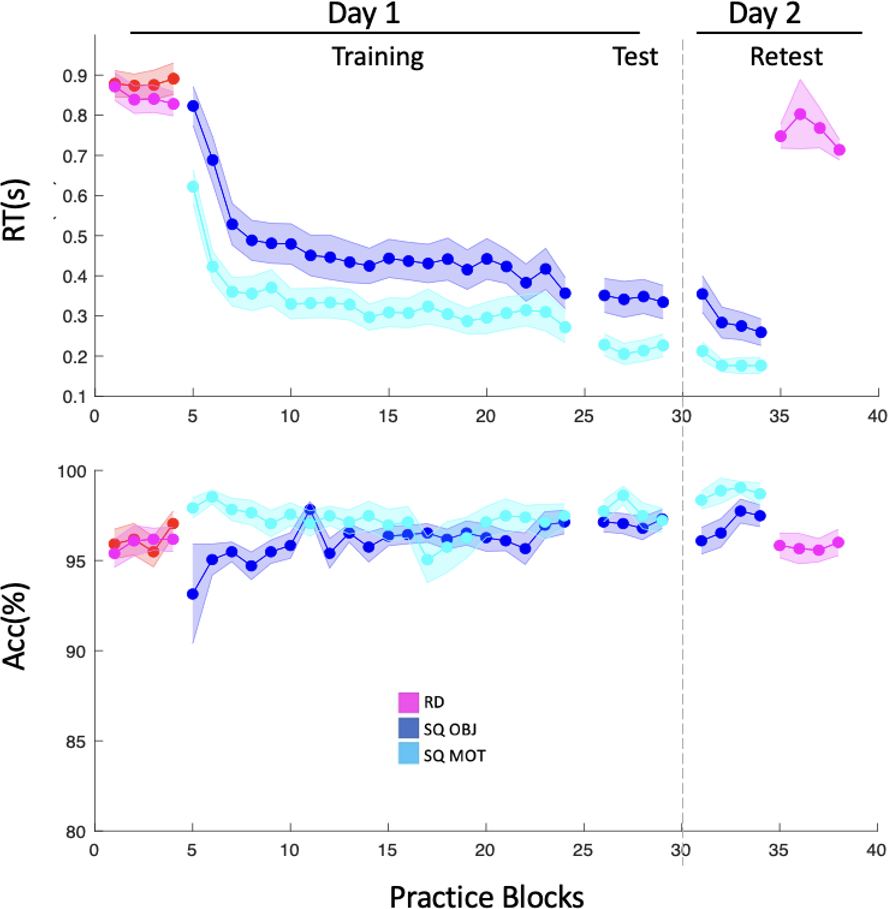

Supplement: S4 Fig — Behavioral results from separate control behavioral experiment. Response time in seconds (top panel) and accuracy (% correct response, bottom panel) across blocks and sessions for all task conditions. Dark blue: object sequence (SQ OBJ), light blue: motor sequence (SQ MOT), pink: random blocks (RD) (darker shade corresponds to random practice during the object session). Shaded error bars = SEM. (n = 24). The source data corresponding to this figure are included in S2 Data, “S4 Fig” sheet. (TIF) [file pbio.3003267.s006.tif]

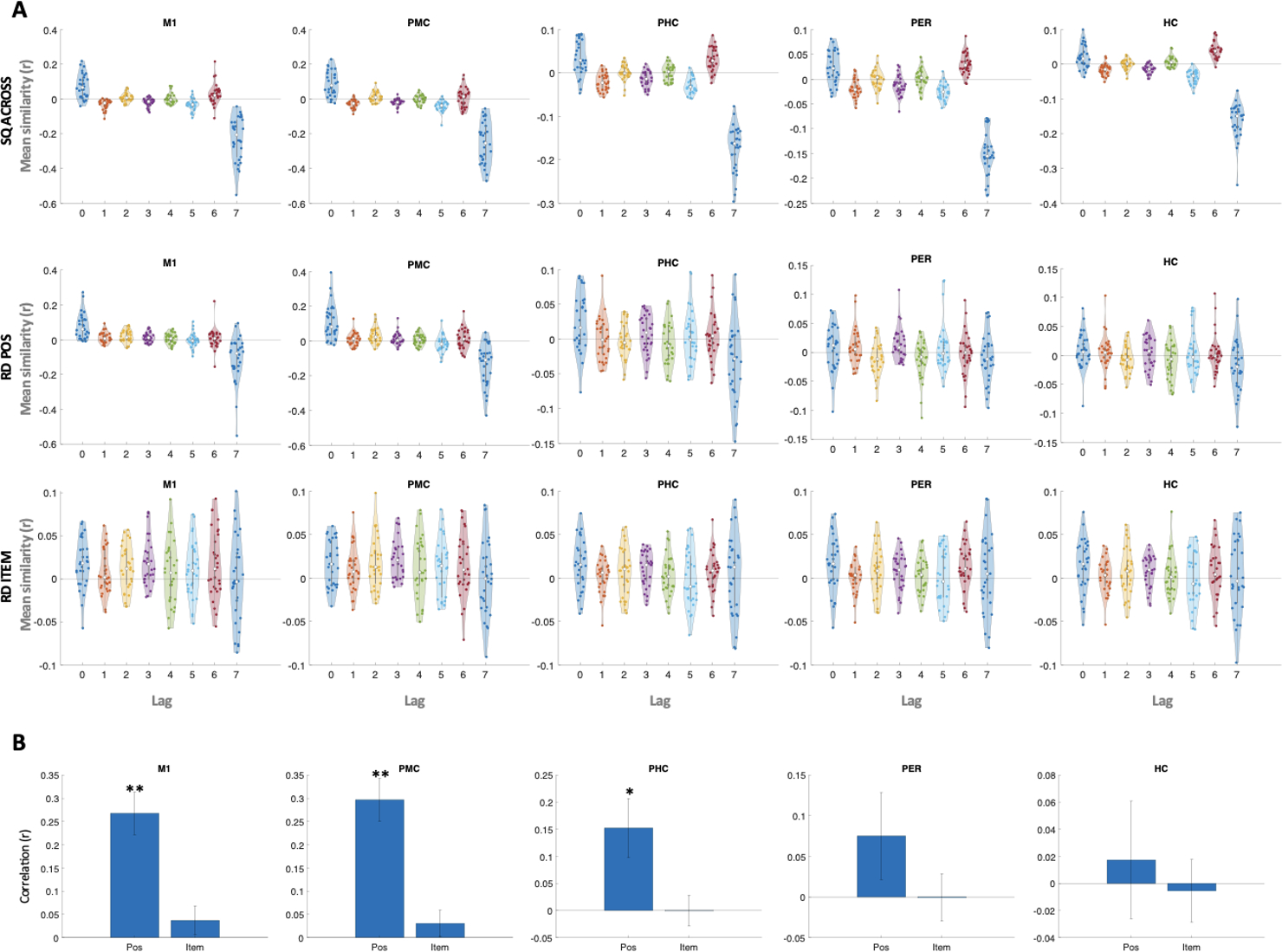

Supplement: S5 Fig — Pattern similarity as a function of lag. (A) Pattern similarity extracted for the across sequences (top), random position (middle) and random item (bottom) matrix as a function of lag in the learned sequences for M1, PMC, PER, PHC and HC. For each ROI, the leftmost bar illustrates mean pattern similarity across repetitions of the same item (key/object) in the same position (“lag 0”). The other bars illustrate pattern similarity across repetitions of items (key/objects) separated by one (“lag 1”), two (“lag 2”), three (“lag 3”) or more (“lag 4”, “lag 5”, “lag 6”, “lag 7”) positions in the learned sequence. SQ ACROSS matrix: Results indicate a main effect of lag in all ROIs (M1: F(7,203) = 60.22, ɳp2 = .68, pcorr < .005; PMC: F(7,203) = 82.54, ɳp2 = .74, pcorr < .005; PHC: F(7,203) = 146, ɳp2 = .83, pcorr < .005; PER: F(7,203) = 140.2, ɳp2 = .83, pcorr < .005; HC: F(7,203) = 161.26, ɳp2 = .85, pcorr < .005). Follow-up analyses indicate that lag 0 was greater than all other lags (all pcorr < .05) except for lag 0 versus lag 6 in M1, PHC, PER and HC (all pcorr = 1) and also between lag 0 versus lag 4 in PER, pcorr = .1 and HC, pcorr = 1). Lag 7 showed lower similarity than all other lags (all pcorr < .05) in all ROIs. In M1 and PMC, a decrease in similarity was observed in odd lags 1, 3 and/or 5 compared to other lags (M1: lags 1, 5 < 2, 6, all pcorr < .05; PMC: lag 5 < 2, 3, 4 and lags 1, 3 < 2, all pcorr < .05). This effect was less pronounced in the PHC, PER and HC, where a decrease in similarity was only observed in lag 5 compared to other lags (lag 5 < 2–4, all pcorr < .05 and lag 5 < 1, in HC only, pcorr < .005). In the HC, an increase in similarity was also observed in even lags 4 and 6 compared to lags 1, 3 and/or 5 (all pcorr < .005). RD POS matrix: Results indicate a main effect of lag in all ROIs except HC and PER (M1: F(7,203) = 19.1, ɳp2 = .4, pcorr < .005; PMC: F(7,203) = 30.3, ɳp2 = .5, pcorr < .005; PHC: F(7,203) = 4.61, ɳp2 = .14, pcorr < .005; HC: F(7, [file pbio.3003267.s007.tif]

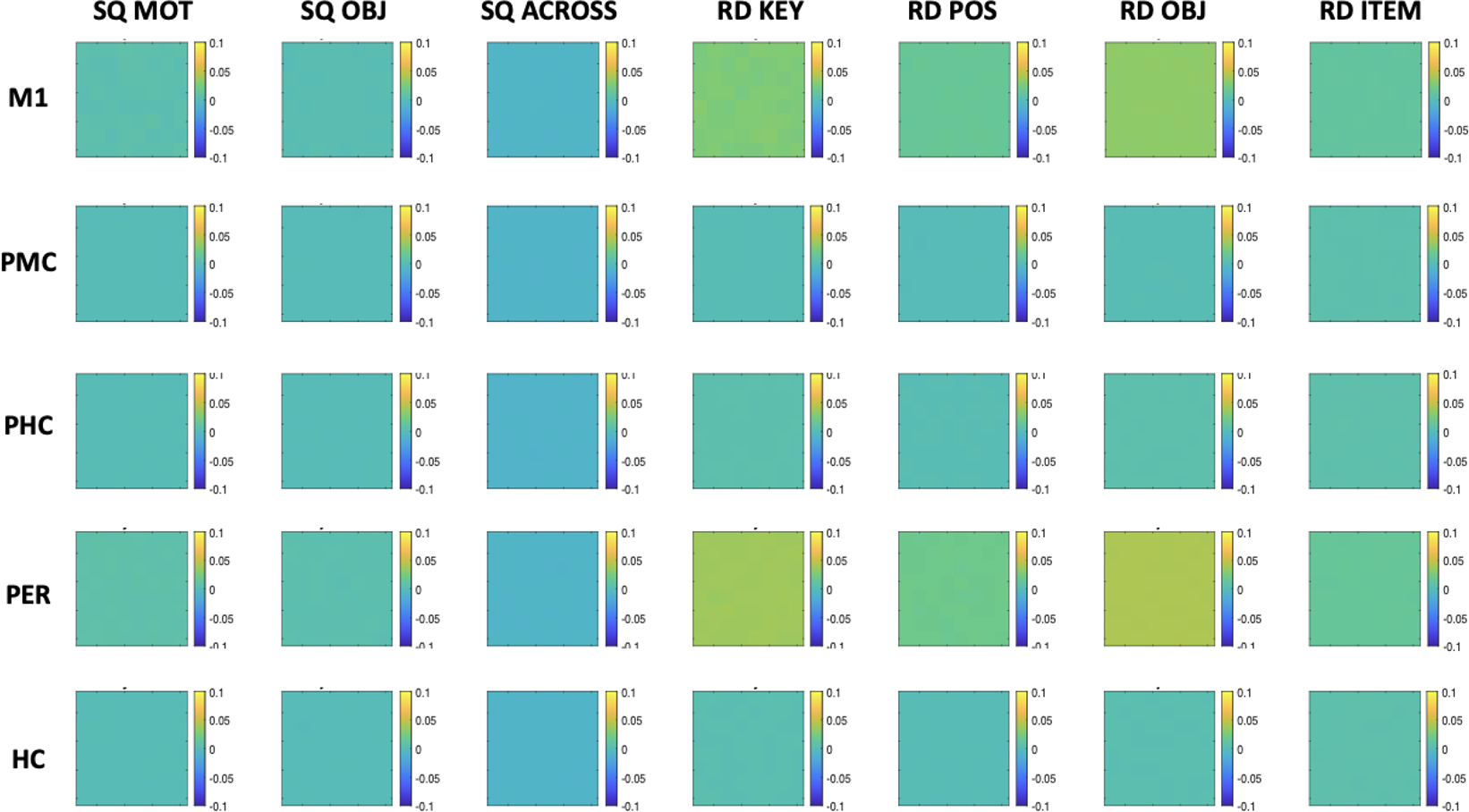

Supplement: S6 Fig — Surrogate matrices by condition (i.e., SQ MOT, SQ OBJ, SQ ACROSS, RD KEY, RD POS, RD OBJ and RD ITEM) for all ROIs. Color bars represent mean similarity (r). The source data corresponding to this figure are included in S2 Data, “S6 Fig” sheet. (TIF) [file pbio.3003267.s008.tif]

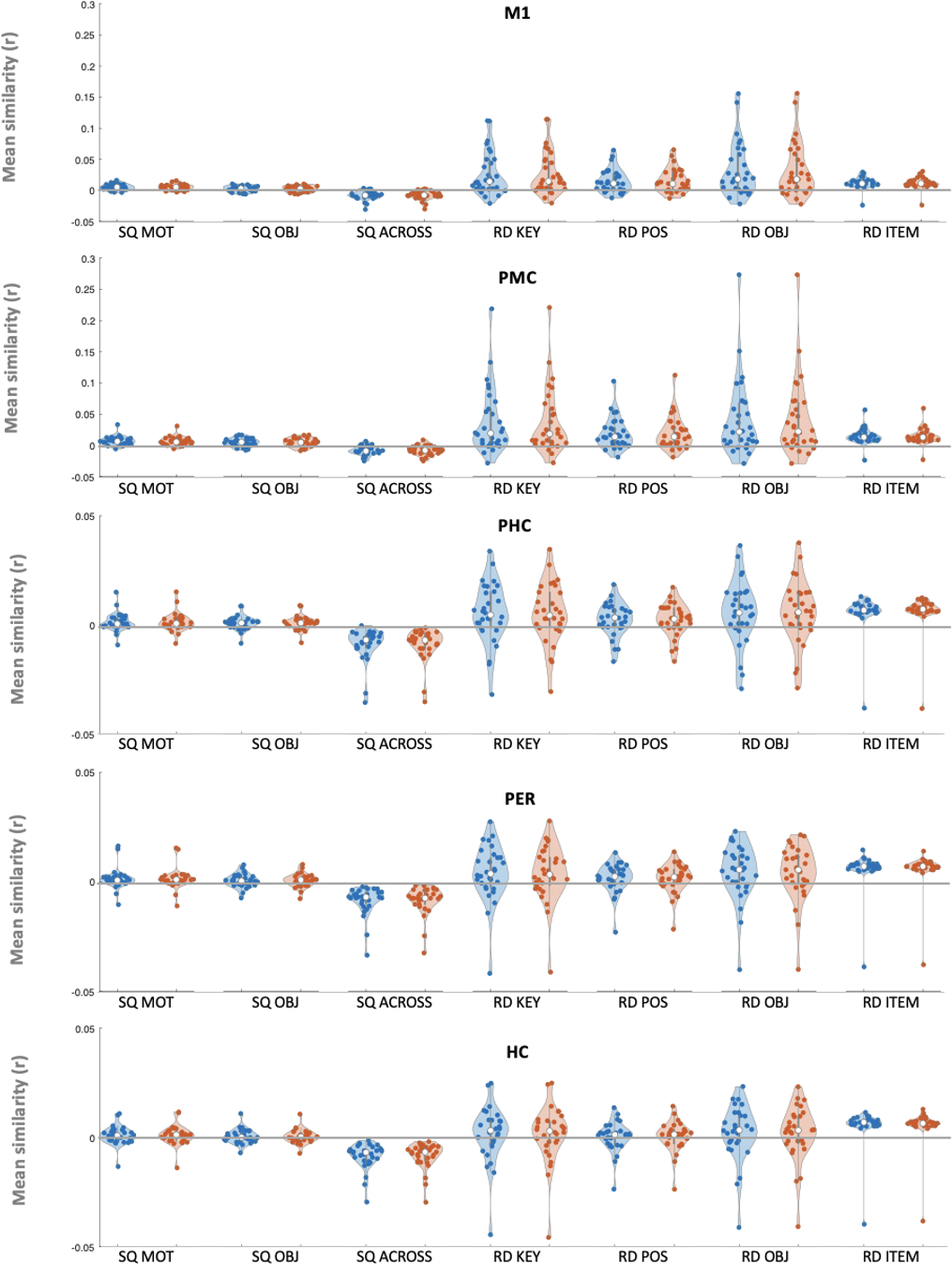

Supplement: S7 Fig — For all ROIs and all condition there were no significant differences in mean pattern similarity between diagonal and off-diagonal cells. Colored circles represent individual data, jittered in arbitrary distances on the x-axis to increase perceptibility and white circles represent medians. The shape of the violin [23] depicts the kernel density estimate of the data. The source data corresponding to this figure are included in S2 Data, “S7 Fig” sheet. (TIF) [file pbio.3003267.s009.tif]

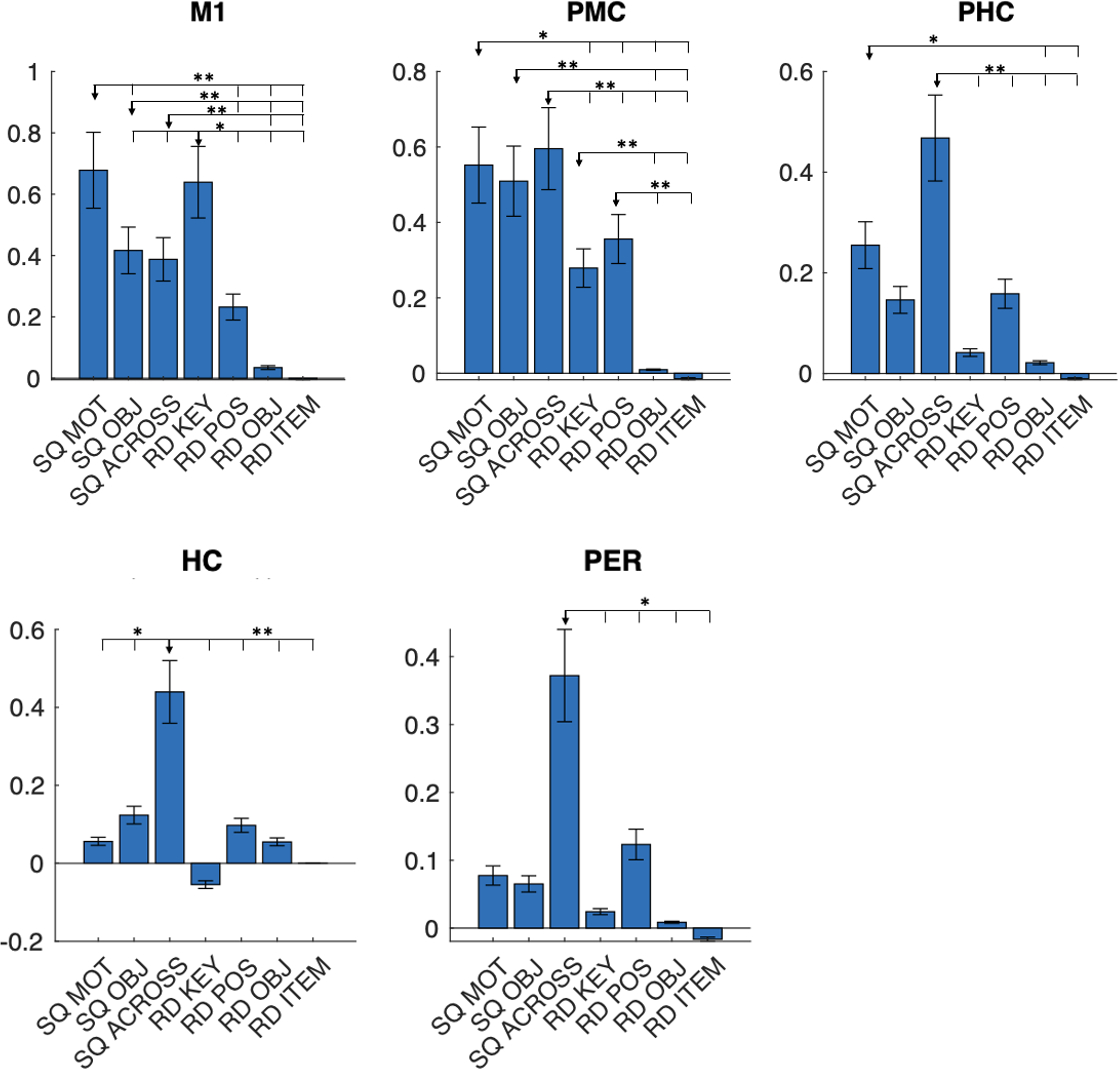

Supplement: S8 Fig — Reliability measures for each condition (i.e., SQ MOT, SQ OBJ, SQ ACROSS, RD KEY, RD POS, RD OBJ and RD ITEM) for each ROI. The measure of reliability was calculated as the similarity between the matrices resulting from a half split cross validation analysis. Higher r values represent higher reliability. Error bars denote ±1 SEM. Arrows indicates the condition that is compared to the other conditions. *pcorr < .05 and **pcorr < .01. The source data corresponding to this figure are included in S2 Data, “S8 Fig” sheet. (TIF) [file pbio.3003267.s010.tif]

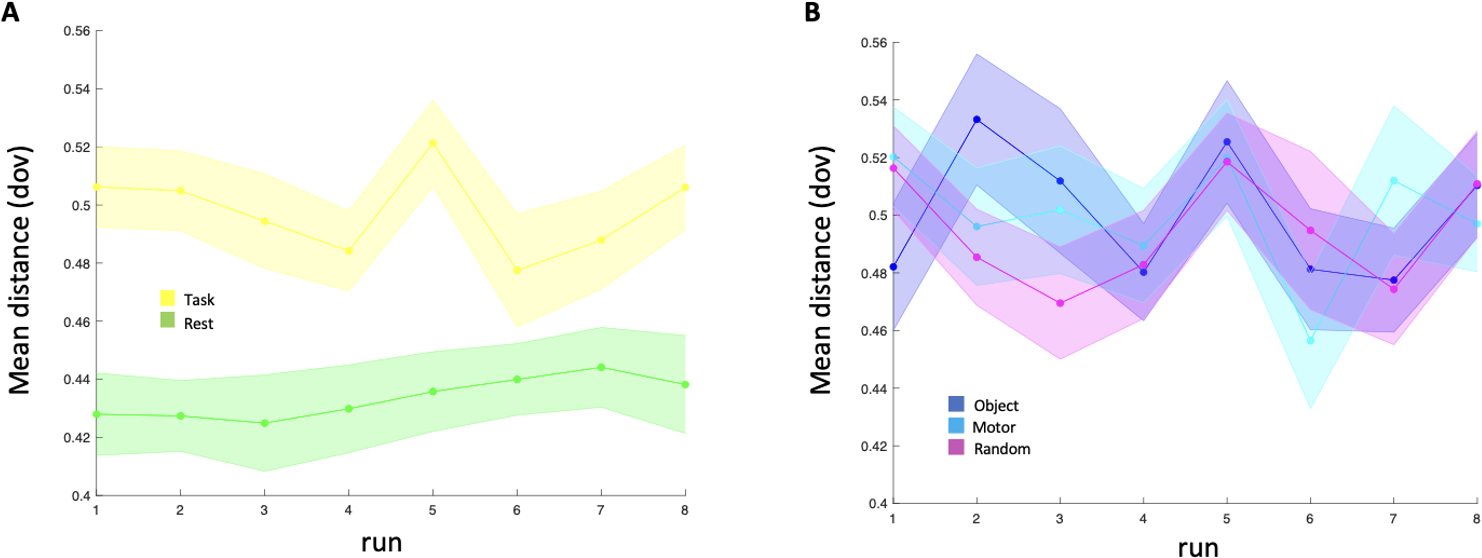

Supplement: S9 Fig — (A) Mean distance (in degrees of visual angle) travelled by the eyes across runs of task (motor, object and random) and runs of rest. (B) Mean distance travelled by the eyes across runs per condition (motor, object and random). Shaded regions denote ±1 SEM. The source data corresponding to this figure are included in S2 Data, “S9 Fig” sheet. (TIF) [file pbio.3003267.s011.tif]

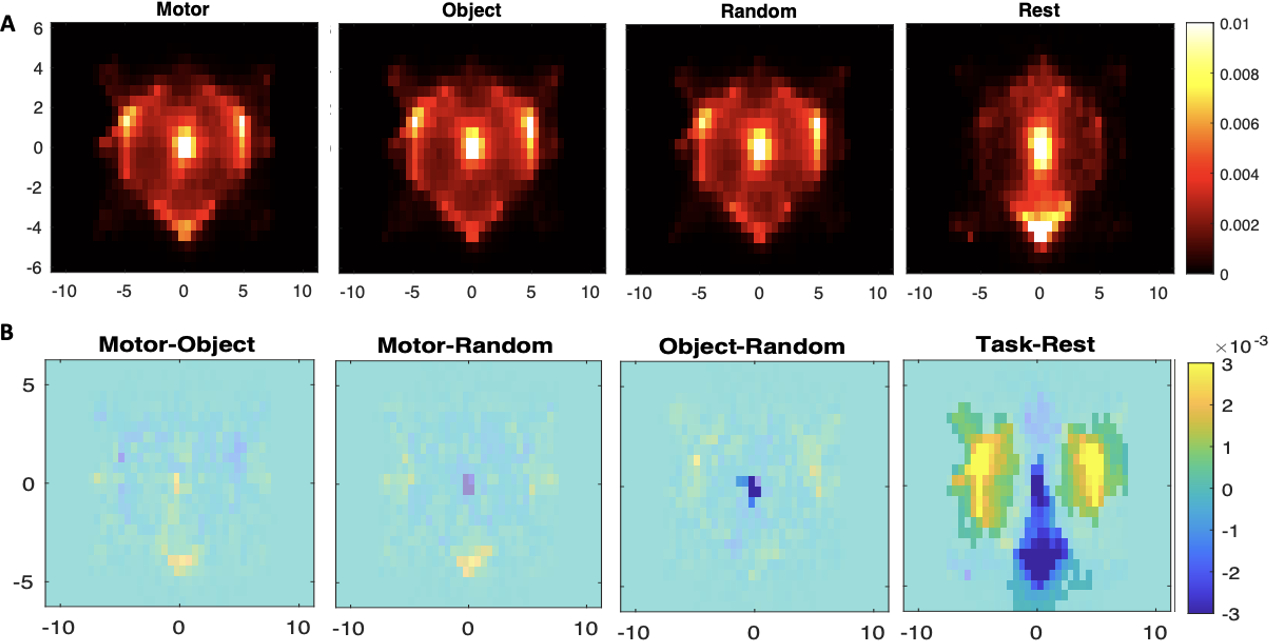

Supplement: S10 Fig — (A) Gaze heatmaps for the motor sequence, object sequence, random and rest conditions averaged across the 8 MRI runs. Brighter values represent longer fixation durations. (B) Differential heat maps between the motor versus object sequence conditions, the motor sequence versus random conditions, the object sequence versus random conditions, and between the task (motor, object and random) versus the rest condition. Clusters representing significant differences in gaze patterns between conditions are highlighted in darker color in the object versus random and task versus rest comparisons. Note that as we did not perform any calibration and pre-trained data was used to extract gaze coordinates, we cannot conclude on the specific gaze location in the different conditions. Accordingly, we only reflect as to whether fixation was more central or lateral (e.g., task versus rest contrast). The source data corresponding to this figure are included in S2 Data, “S10 Fig” sheet. (TIF) [file pbio.3003267.s012.tif]

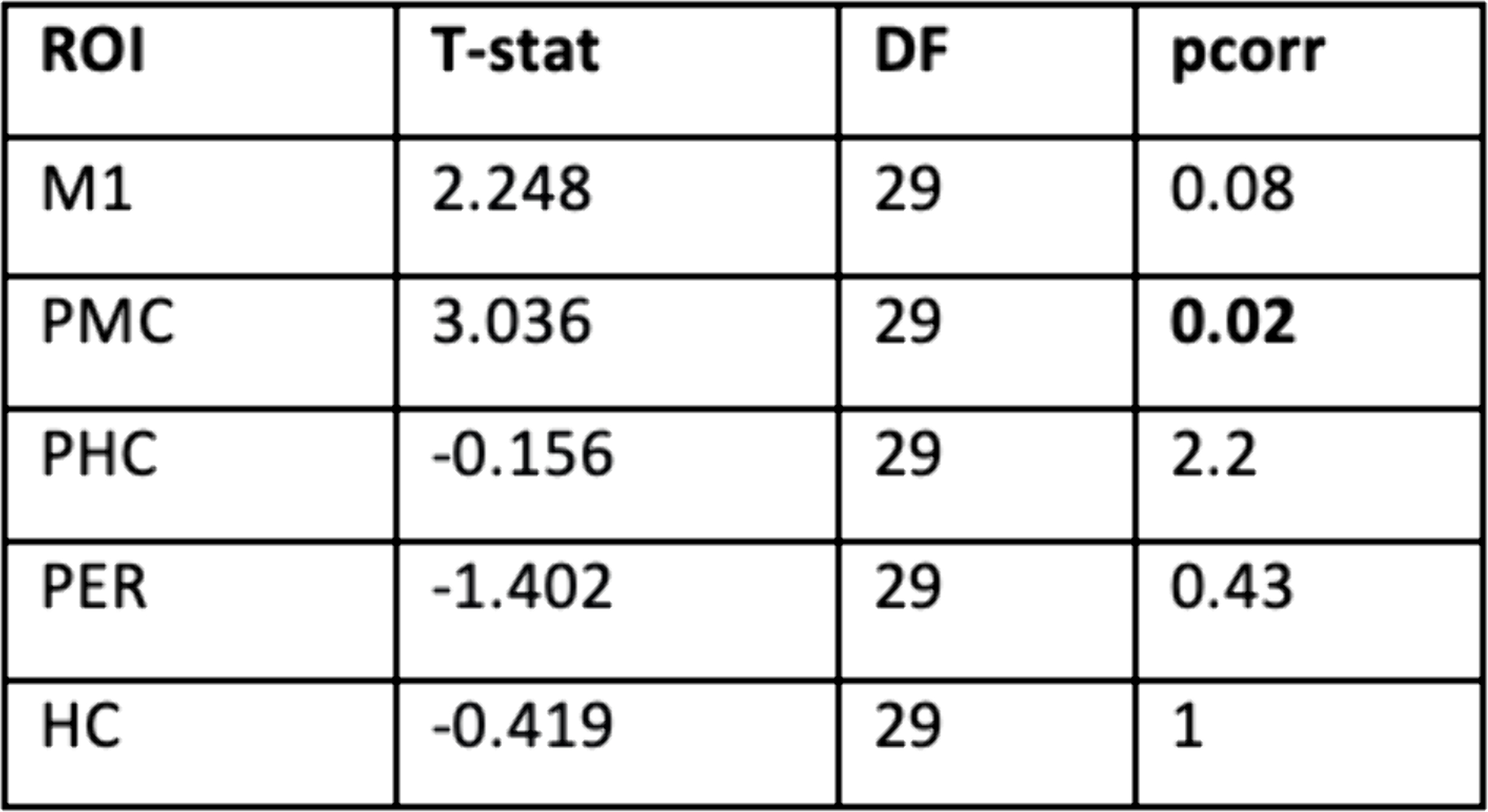

Supplement: S1 Table — One-tailed one sample-test results for the comparison of delta similarity (mean diagonal cells versus mean off-diagonal cells) against zero for position matrices after edge removal (6 × 6 matrices). After removing boundary positions, position coding only remained significant in the premotor cortex while such coding was no longer significant in all other ROIs. P values are Bonferroni-corrected. The source data used to perform the statistical tests reported in this table are included in S2 Data, “S1 Table” sheet. (TIF) [file pbio.3003267.s013.tif]

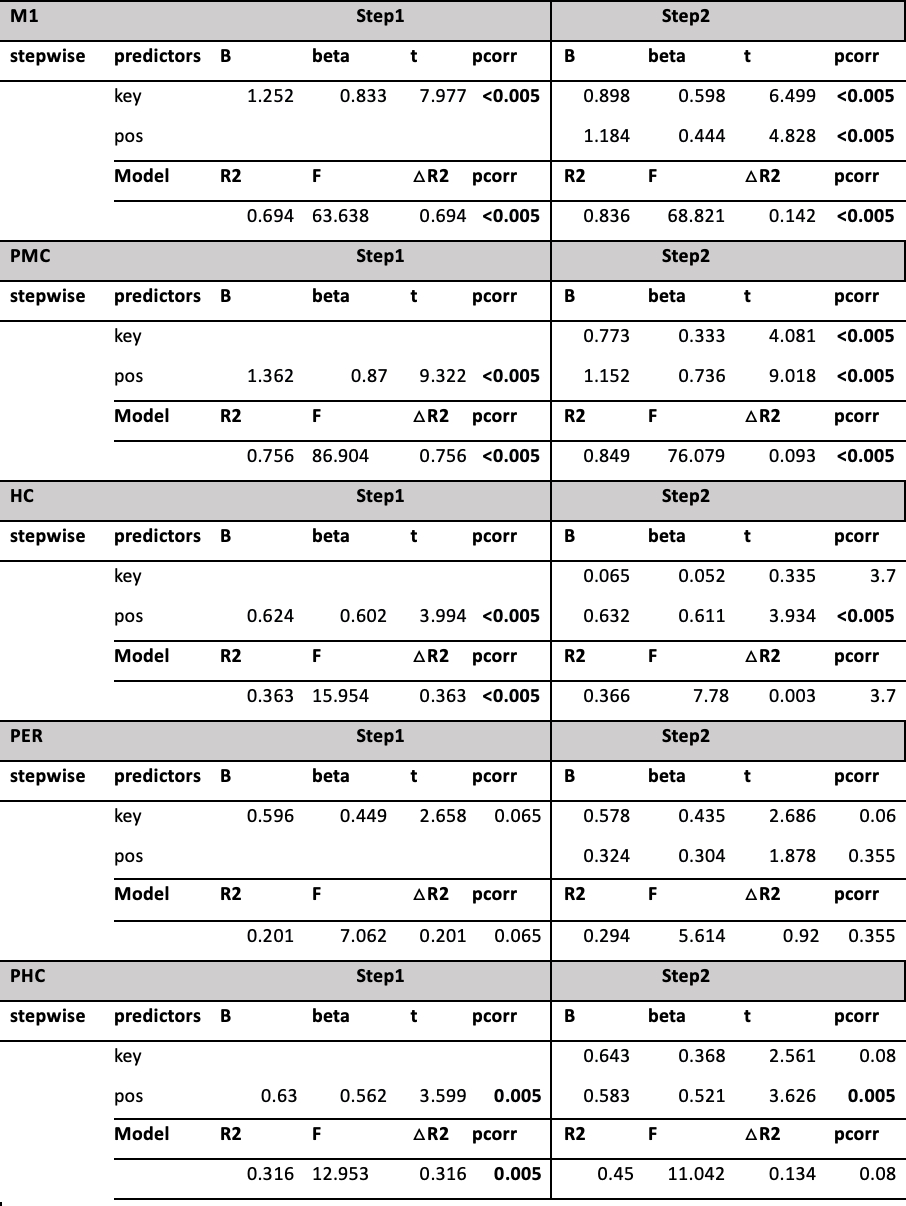

Supplement: S2 Table — Dependent variable: delta similarity (i.e., difference between mean diagonal and mean off-diagonal cells) in the motor sequence matrix. Predictors: delta similarity in the random key and/or position matrix. An F-test was used to assess whether the change in explained variance (ΔR2) from the prior step is significant. Step 1, df1 = 1, df2 = 28; Step 2, df1 = 2, df2 = 27. P-values are Bonferroni corrected. The source data used to perform the statistical tests reported in this table are included in S2 Data, “S2 Fig” sheet. (TIF) [file pbio.3003267.s014.tif]

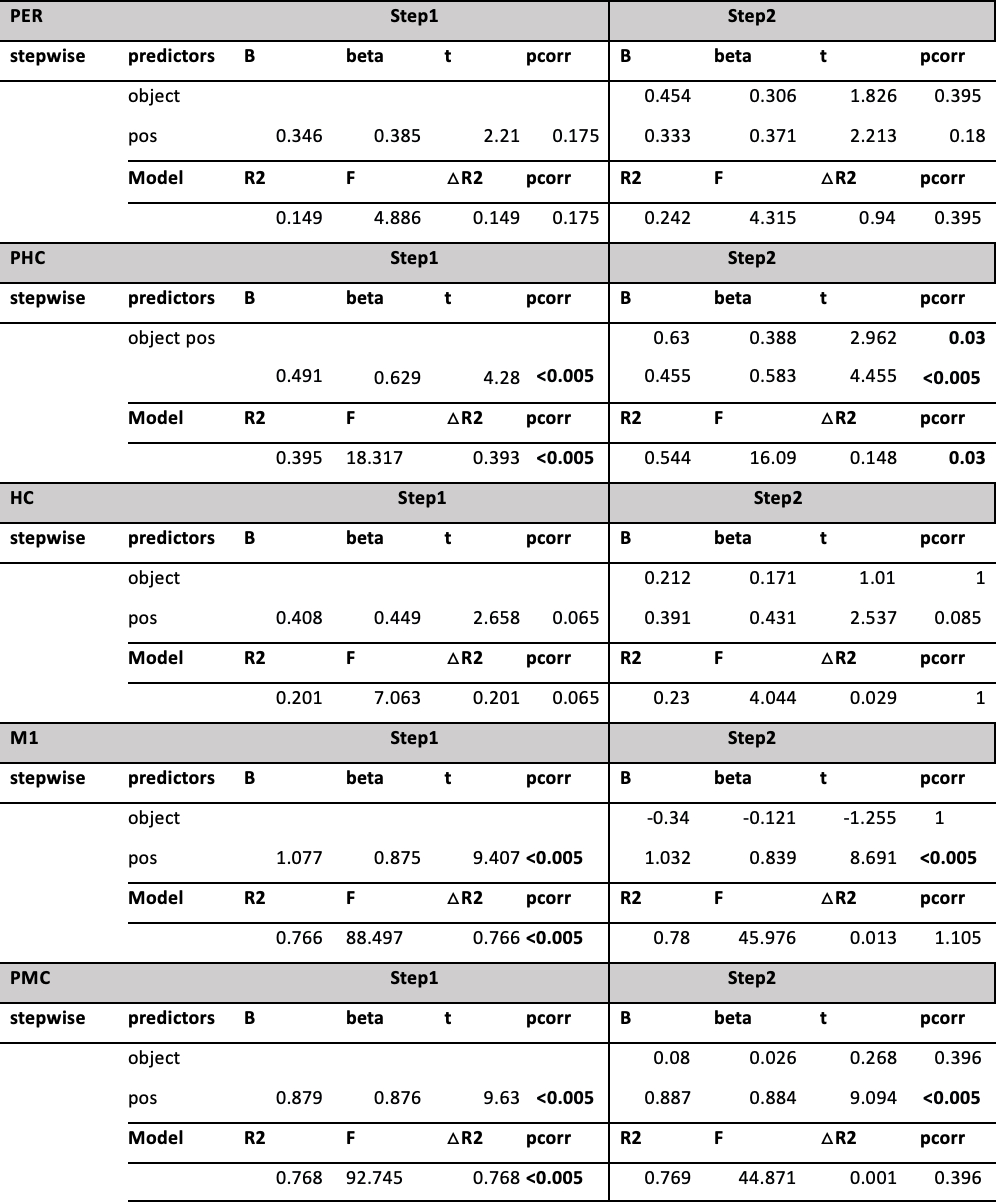

Supplement: S3 Table — Dependent variable: delta similarity (i.e., difference between mean diagonal and mean off-diagonal cells) in the object sequence matrix. Predictors: delta similarity in the random object and/or position matrix. An F-test was used to assess whether the change in explained variance (ΔR2) from the prior step is significant. Step 1, df1 = 1, df2 = 28; Step 2, df1 = 2, df2 = 27. P-values are Bonferroni corrected. The source data used to perform the statistical tests reported in this table are included in S2 Data, “S2 Fig” sheet. (TIF) [file pbio.3003267.s015.tif]

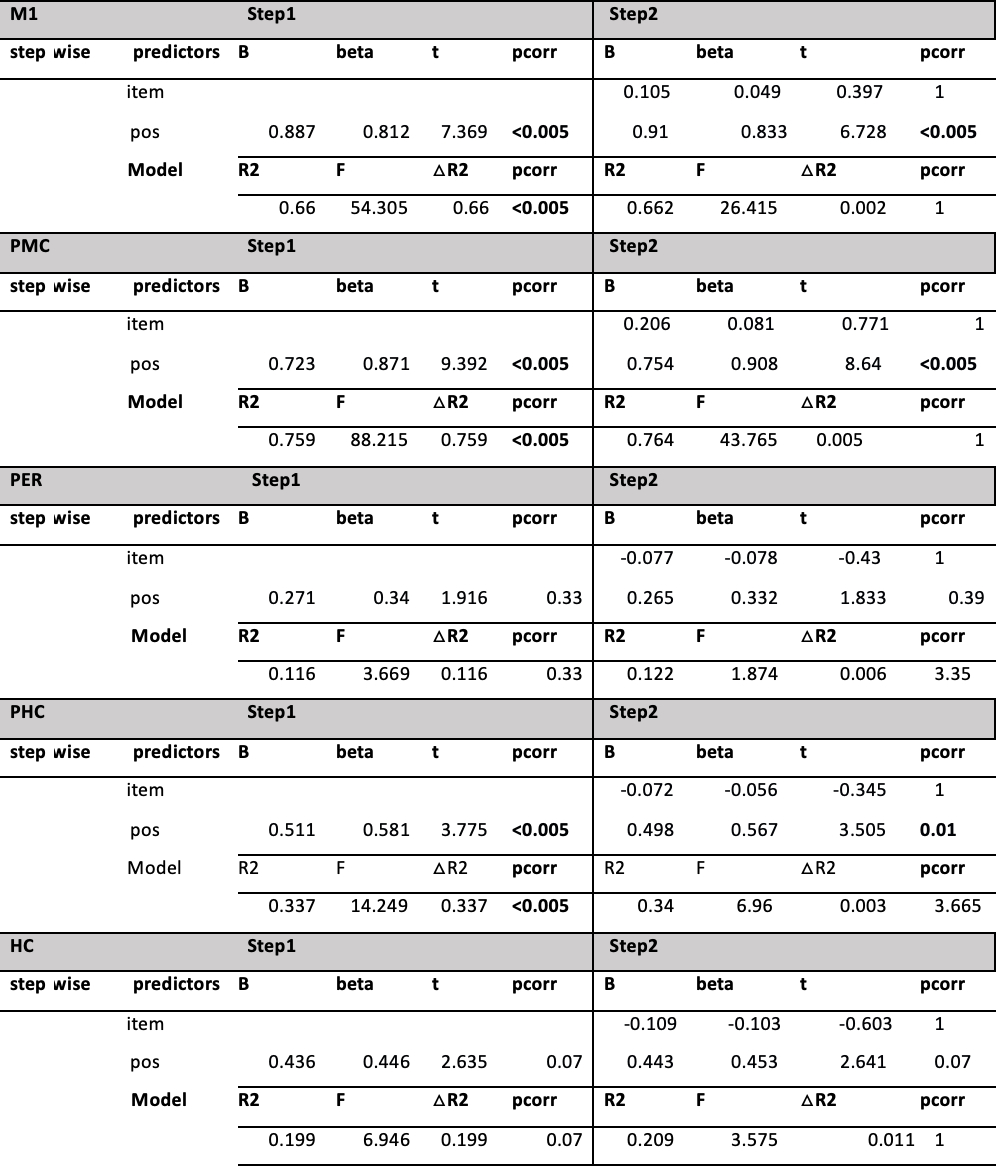

Supplement: S4 Table — Dependent variable: delta similarity (i.e., difference between mean diagonal and mean off-diagonal cells) in the item-position matrix. Predictors: delta similarity in the random item and/or position matrix. An F-test was used to assess whether the change in explained variance (ΔR2) from the prior step is significant. Step 1, df1 = 1, df2 = 28; Step 2, df1 = 2, df2 = 27. P-values are Bonferroni corrected. The source data used to perform the statistical tests reported in this table are included in S2 Data, “S2 Fig” sheet. (TIF) [file pbio.3003267.s016.tif]

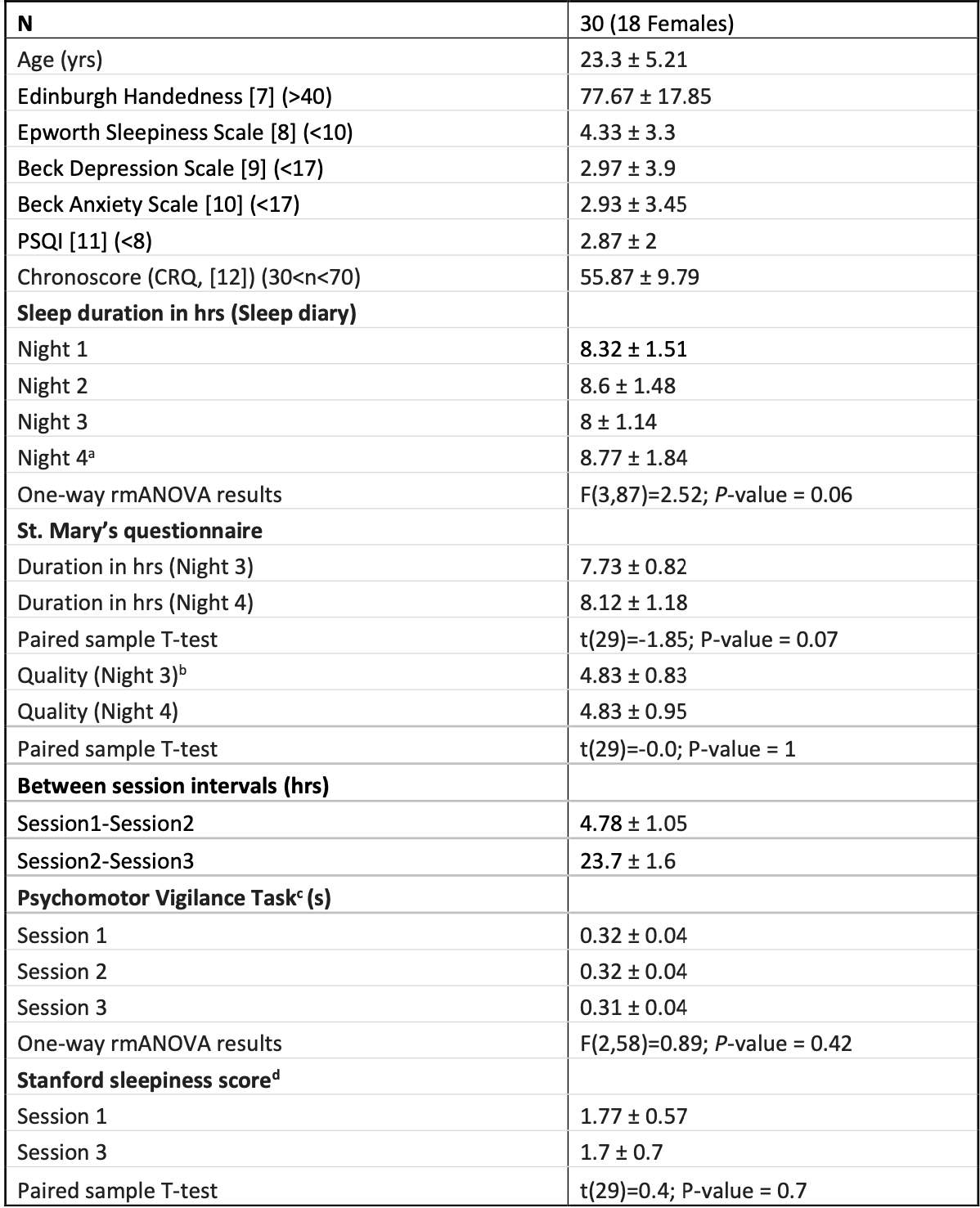

Supplement: S5 Table — Participant demographics, sleep and vigilance characteristics. Notes. Values are means and standard deviations (unless noted otherwise). Cutoff values for exclusion in parentheses. PSQI = Pittsburgh Sleep Quality Index. CRQ = Circadian Rhythm Questionnaire. aDetermined in combination between sleep diary and wrist actigraphy recordings on night 4 (between the two experimental sessions). b1 = very badly, 6 very well. cMedian of reaction times. d1 = more alert, 7 = less alert. The source data corresponding to this table are included in S2 Data, “S5 Table” sheet. (TIF) [file pbio.3003267.s017.tif]

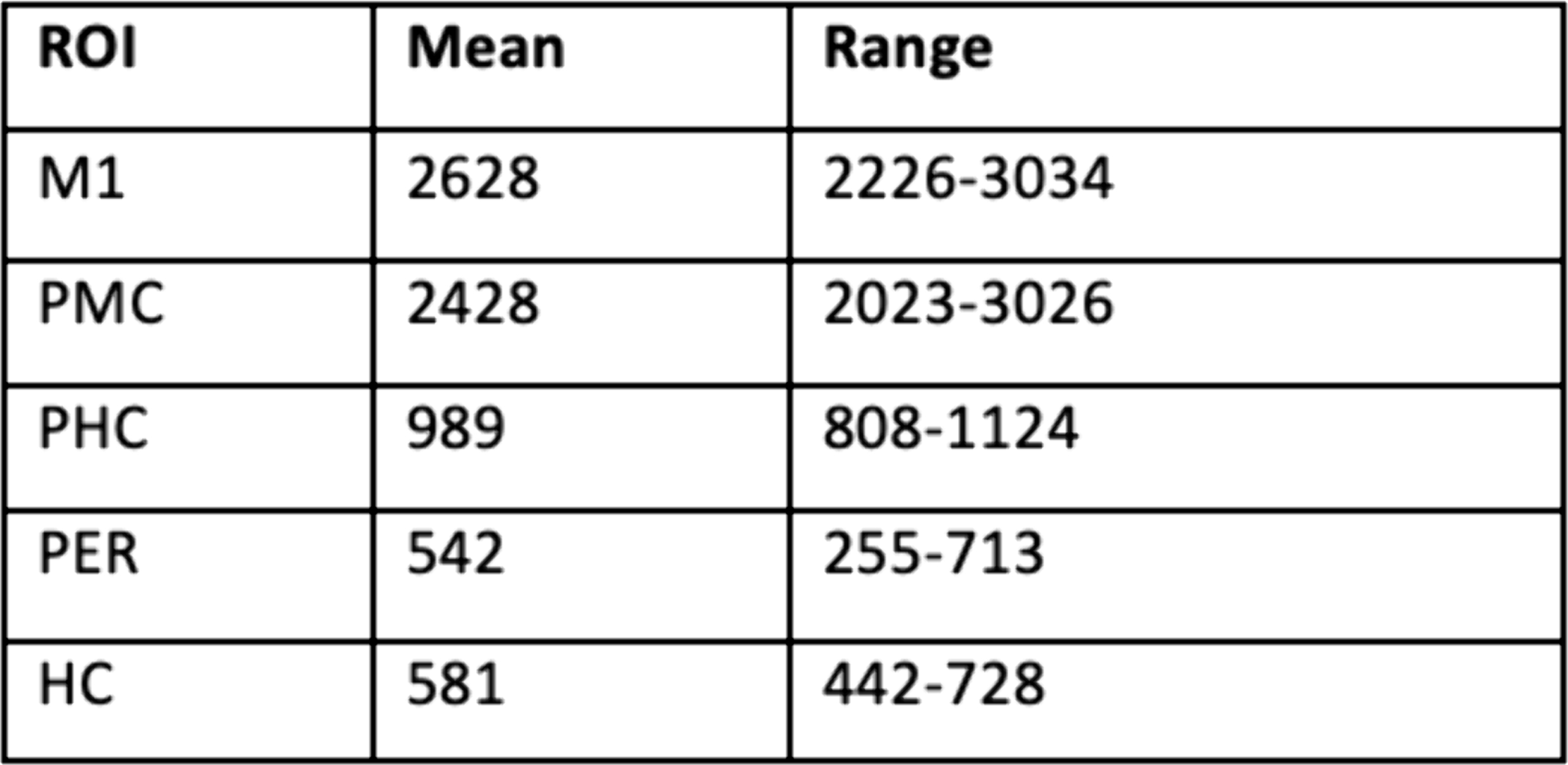

Supplement: S6 Table — ROI size (number of voxels). The source data corresponding to this table are included in S2 Data, “S6 Table” sheet. (TIF) [file pbio.3003267.s018.tif]

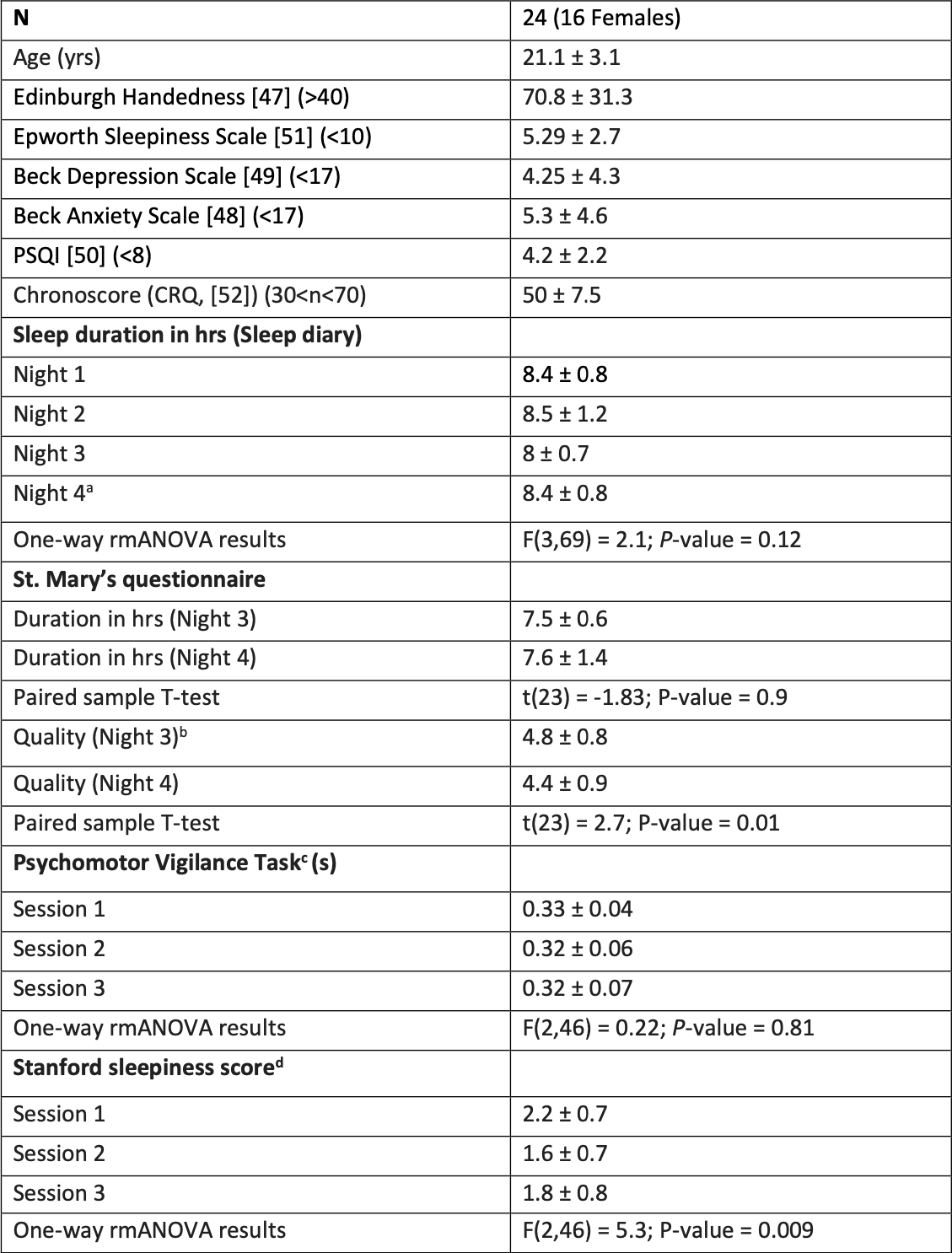

Supplement: S7 Table — Participant demographics, sleep and vigilance characteristics for Control behavioral experiment. Notes. Values are means and standard deviations (unless noted otherwise). Cutoff values for exclusion in parentheses. PSQI = Pittsburgh Sleep Quality Index. CRQ = Circadian Rhythm Questionnaire. aDetermined in combination between sleep diary and wrist actigraphy recordings on night 4 (between the two experimental sessions). b1 = very badly, 6 very well. cMedian of reaction times. d1 = more alert, 7 = less alert. The source data corresponding to this table are included in S2 Data, “S7 Table” sheet. (TIF) [file pbio.3003267.s019.tif]
