# Supplementary material for: The human medial temporal lobe represents memory items in their ordinal position in both declarative and motor memory domains
Source: PLoS Biol. 2025 Jul 7;23(7):e3003267. doi: 10.1371/journal.pbio.3003267 (PMC12258568; doi:10.1371/journal.pbio.3003267)
Supplement: S1 Text — (DOCX) [file pbio.3003267.s020.docx]

Supplementary analyses

1. Assessment of baseline performance

We tested whether general motor execution, assessed with the random SRTT, differed at baseline between the 2 learning sessions on day 1 (see Fig 1A in the main text). Results show that performance speed (i.e., mean response time) increased across blocks of the random SRTT (block effect: F(3,87)=7.71, ɳ_p_^2^=.21, p<.001) but this effect did not differ between sessions (session by block effect: F(3,87)=.64, ɳ_p_^2^=.02, p=.59; session effect F(1,29)=.02, ɳ_p_^2^=.001, p=.89; see upper panel of Fig 2 in the main text). Performance accuracy (i.e., % correct responses) remained stable across blocks of the random SRTT (session by block effect: F(3,87)=.39, ɳ_p_^2^=.01, p=.76; block effect: F(3,87)=1.07, ɳ_p_^2^=.04, p=.37; see bottom panel of Fig 2 in the main text) but was better during the object task session (session effect; F(1,29)=5.9, ɳ_p_^2^=.17, p=.02).

1. Session order effects on performance

Even though condition order was counterbalanced across participants on experimental days 1 (learning) and 2 (retest), we tested whether order influenced performance on both the random and sequence SRTT. Results of the ANOVAs using session order, irrespective of task condition and block as within-subject factors are presented below.

Day 1 data

For the random SRTT, performance speed increased across blocks (block effect: F(3,87)=7.71, ɳ_p_^2^=.21, p<.001) and was overall better in session 2 than in session1 (session by block effect: F(3,87)=8.86, ɳ_p_^2^=.23, p<.001; session effect F(1,29)=87.23 ɳ_p_^2^=.75, p<.01). In contrast, accuracy remained stable across blocks of the random SRTT (block effect: F(3,87)=1.07, ɳ_p_^2^=.04, p=.37) and similar across sessions (session effect; F(1,29)=.41, ɳ_p_^2^=.01, p=.53; session by block effect: F(3,87)=1, ɳ_p_^2^=.03, p=.4).

For the sequential SRTT, performance speed improved during training in both sessions (block effect; training: F(19,551)=38.98, ɳ_p_^2^=.57, p<.001; test: F(3,87)=3.78, ɳ_p_^2^=.12, p=.01) and the block by session interaction was significant such that block-to-block changes in performance were steeper in the early blocks of session 2 as compared to session 1 (condition by block effect; training: F(19,551)=4.92, ɳ_p_^2^= .15, p<.001; note that this effect was no longer observed during test: F(3,87)=.33, ɳ_p_^2^=.01, p=.8). However, overall performance speed did not differ between sessions (session effect; training: F(1,29)=.95, ɳ_p_^2^=.03, p=.34; test: F(1,29)=.58, ɳ_p_^2^= .02, p=.45). Performance accuracy remained similar across sessions (training: session by block effect: F(19,551)=1.64, ɳ_p_^2^=.05, p=.04; block effect: F(19,551)=1.43, ɳ_p_^2^=.05, p=.11; session effect: F(1,29)=.02, ɳ_p_^2^=.001, p=.89; test: session by block effect: F(3,87)=1.96, ɳ_p_^2^=.06, p=.13; block effect F(3,87)=.72, ɳ_p_^2^=.02, p=.55; session effect: F(1,29)=.19, ɳ_p_^2^=.01, p=.66).

Day 2 data

During the retest session on day 2, performance speed improved across practice blocks while accuracy remained stable (block effect; speed: F(3,87)=9.07, ɳ_p_^2^=.24, p<.001; accuracy: F(3,87)=1.70, ɳ_p_^2^=.06, p=.17). Performance speed was faster for the task that was retested second, while accuracy was similar between sessions (order effect; speed: F(1,29)=6.65; ɳ_p_^2^=.19, p=.02; accuracy: F(1,29)=.05, ɳ_p_^2^=.002, p=.83)

Overall, these results suggest that on day 1, participants tended to be faster at the beginning of session 2 as compared to session 1 (random task and early blocks of sequence tasks) which suggests skill transfer between sessions. An order effect was also observed on day 2 during retest outside the scanner. However, it is unlikely that these order effects influenced our results as the order of the conditions was counterbalanced across participants (15 participants started with the motor condition and 15 participants started with the object condition).

1. Separate control behavioral experiment

To better characterize sequence learning in this new paradigm, we collected data from an independent sample of participants with a design that closely mirrored the one used in the current study but with the addition of generation tasks similar as in earlier research [1,2]. Specifically, the object generation/recall tasks aimed at reconstructing the temporal order of the object items and the motor generation task tested for any explicit knowledge of the learned sequences of movements. Note that minor modifications were also made to the design of this control experiment in order to address an additional research question that is not related to the current research (see task and procedure below).

Participants

24 young (aged 18-35) healthy adults were recruited for this study using the same recruitment and screening procedure and the same inclusion criteria as in the current study – with two exceptions: left-handed participants were included (n = 1), and participants were not required to meet the MRI safety criteria. Participant demographics as well as information on their sleep and vigilance are presented in S7 Table.

Task

The task design was identical to the current study with the following exceptions: 1) the task was unimanual rather than bimanual and was performed with the non-dominant hand. The task consisted of a 4-choice reaction time task (8-element sequence), where 8 different objects were constantly presented on the bottom of the screen in 2 rows of 4 objects. Each column of objects was mapped to one of the four fingers used to perform the task; 2) participants learned one of two motor sequences (seq A: 4,1,3,2,4,3,1,2 and seq B: 2,3,1,4,3,2,4,1) and one of two object sequences (seq A: car, pineapple, giraffe, saw plane, elephant, banana axe and seq B: saw, giraffe, pineapple, car, elephant, axe, plane, banana); 3) The response-stimulus interval (RSI) was slightly longer (1s rather as opposed to 750ms in the current study); 4) A generation/recall task was employed to confirm that participants developed an explicit knowledge of the series of objects and movements with this paradigm. The generation/recall task consisted of a free recall test. For the object generation/recall task, participants were instructed to verbally recall the series of objects learned in the correct order, at their own pace, three times in a row. Performance was audio-recorded for further analyses. For the motor generation task, participants were instructed to generate on the keyboard, at their own pace, the learned sequence of finger movements three times in a row. Performance from the keyboard was logged for further analyses. The order of the object and motor sequence generation tasks was counterbalanced across participants.

Procedure

The procedure was identical to the current study except that there was no MRI session on Day 2 in this control experiment. Day 2 included only the retest session to assess retention, followed by the sequence generation task to assess explicit knowledge of the motor and object sequences.

Results

*Performance speed & accuracy*

The behavioral results of this separate experiment closely mirrored the results of the current study (see S4 Fig). Specifically, we tested whether general motor execution, assessed with the random SRTT, differed at baseline between the 2 learning sessions on day 1. Results show that performance speed (i.e., mean response time) and accuracy (i.e., % correct responses) remained stable across blocks of the random SRTT (block effect; speed: F(3,141)=.71, ɳ_p_^2^=.02, p=.55; accuracy: F(3,141)=.89, ɳ_p_^2^=.02, p=.45) and did not differ between conditions (condition by block effect; speed: F(3,141)=1.7, ɳ_p_^2^=.04, p=.17; accuracy F(3,141)=1.8, ɳ_p_^2^=.04, p=.15; condition effect; speed: F(1,47)=.95, ɳ_p_^2^=.02, p=.33; accuracy: F(1,47)=.06, ɳ_p_^2^=.001, p=.82). Analyses of the sequence SRTT data indicated that performance speed (i.e., mean response time) improved during learning on day 1 for both the motor and object sequence tasks (block effect; F(19,893)=48.7, ɳ_p_^2^=.51, p<.001) but the motor task presented overall faster performance (condition by block effect; F(19,893)=2.63, ɳ_p_^2^= .05, p<.001; condition effect; F(1,47)=29.1, ɳ_p_^2^=.38, p<.001; S4 Fig, day 1). Performance speed stabilized during the test blocks (block effect; F(3,141)=1.3, ɳ_p_^2^=.03, p=.27), but remained faster for motor compared to object (condition by block effect; F(3,141)=1.8, ɳ_p_^2^=.04, p=.2; condition effect; F(1,47)=30.6, ɳ_p_^2^=.4, p<.001). In contrast, performance accuracy remained stable during learning on day 1 and was slightly higher for the motor task compared to the object task during training but then became similar between tasks at test (training: condition by block effect: F(19,893)=2.2, ɳ_p_^2^=.05, p=.002; block effect: F(19,893)=.8, ɳ_p_^2^=.02, p=.71; condition effect: F(1,47)=15.5, ɳ_p_^2^=.25, p<.001; test: condition by block effect: F(3,141)=.65, ɳ_p_^2^=.01, p=.58; block effect F(3,141)=1.69, ɳ_p_^2^=.04, p=.17; condition effect: F(1,47)=3.6, ɳ_p_^2^=.07, p=.06).

Data collected on day 2 during the retest, performance speed improved across practice blocks (block effect: F(3,141)=4.9, ɳ_p_^2^=.1, p=.003) while accuracy remained stable (block effect: F(3,141)=2.03, ɳ_p_^2^=.04, p=.11). Importantly, performance was overall better for sequence conditions when compared to random (condition effect; speed: F(2,94)=213.9; ɳ_p_^2^=.82, p<.001; motor vs. random: p<.001; object vs. random: p<.001; accuracy: condition effect; F(2,94)=310.4, ɳ_p_^2^=.4, p<.001; motor vs. random: p<.001; object vs. random: p=.001; S4 Fig, day 2) and performance for the motor task remained better than for the object task (motor vs. object; speed & accuracy: p<.001). Overall, these results suggest that participants specifically learned and retained both the motor and object sequences, although to a different extent as performance was better on the motor as compared to the object task. This suggests that despite the few differences in task design described above, behavior on both tasks was similar between studies.

*Generation*

Analyses of the generation tasks demonstrated that participants successfully reproduced the motor and object sequences with high accuracy. Specifically, the mean percentage of correct ordinal positions, i.e., the percentage of items (movements or objects) generated in the correct temporal position in the sequence and the percentage of correct transitions generated was greater than 89% across tasks (% correct ordinal Mot: 95% (12.1), Obj: 89% (26.2); % correct transitions Mot: 94% (13.3), Obj: 91% (22.8)). A two-tailed paired sample t-tests revealed no significant difference between object and motor sequence recollection in terms of both correct ordinal positions (t(47)=1.78, p=.08) and correct transitions (t(47)=1.29, p=.2). The results from this separate experiment complement the response-based evidence from the SRTT and indicate that participants developed explicit knowledge of the series of objects (and movements) in this paradigm.

1. Brain-behavioral correlation

Since performance differed between conditions during initial learning, we examined whether the level of performance reached at the end of the training session on day 1 (average reaction time in post-training test blocks) was correlated with neural pattern similarity (delta similarity for object-position and finger-position coding) examined the next day. We did not observe any correlation between performance on the motor sequence task and the finger-position coding effect in any of the motor ROIs (M1: r=-.38, p_corr_=.2; PMC: r=-.45, p_corr_=.06; HC: r=-.32, p_corr_=.42) or between performance on the object sequence task and the object-position coding effect in any of the object ROIs (PHC: r=-.27, p_corr_=.73; PER: r=-.28, p_corr_=.68; HC: r=-.2, p_corr_=1). Altogether, these results indicate that the level of performance reached at the end of training on day 1 was not related to the multivoxel activation patterns examined the next day.

1. Lag analyses

In order to assess whether similarity changes depending on the lag between items, we performed similar analyses as in our previous research [3] and examined how pattern similarity changed depending on the lag between items in a learned sequence across memory domains. To do so, pattern similarity for each ROI was extracted from the across domain sequence matrix (**SQ ACROSS**) and averaged across cells as a function of the lags present in the learned sequence. A repeated measures ANOVA was performed on pattern similarity with lag as within-subjects factor (i.e., factor with 8 levels: lag 0 to lag 7). All the results reported below are Bonferroni corrected for multiple comparisons (i.e., main effects corrected for the number of ROIs tested; N=5 tests; pairwise comparisons corrected for the number of comparisons, N=7) and for non-sphericity if applicable (Greenhouse-Geisser correction).

Results are depicted in S5A Fig. Overall, the results do not point to a progressive decrease in similarity with lag as in earlier research [3] but they indicate greater dissimilarity between consecutive items (and items separated by odd lags in general) which is partly in line with our earlier observations [3]. Specifically, the analyses revealed a main effect of lag on pattern similarity in the across sequence matrix in all ROIs (**SQ ACROSS**; top row in S5A Fig; results of the statistical analyses are reported in the caption). In line with the results presented in the main text, planned pairwise comparisons showed that the diagonal (i.e., lag 0, same key/object + position) presented higher similarity values than the large majority of the other lags in all ROIs (corresponding statistics reported in the caption of S5 Fig). In addition, similarity values in lag 7 were significantly lower than all other lags in all ROIs (all *p_corr_*<.005), and this effect was particularly pronounced between lag 0 and lag 7 which suggests large dissimilarities between the first and the last items in the sequence, as observed in our prior work [3]. Interestingly, a decrease in similarity was observed in odd lags across all ROIs (note though that this effect was more pronounced in M1 and PMC, see detailed statistics in the caption of S5A Fig). Exploratory analyses on odd and even lags indicated that pattern similarity was overall significantly higher for even lags (i.e., lags 2, 4 and 6) when compared to odd lags (i.e., lags 1, 3 and 5) in all ROIs (M1: t_(29)_=4.9, *p_corr_*<.005; PMC: t_(29)_=5.3, *p_corr_*<.005; PHC: t_(29)_=6.8, *p_corr_*<.005; PER: t_(29)_=6, *p_corr_*<.005; HC: t_(29)_=9.6, *p_corr_*<.005). Overall, these results indicate greater dissimilarity between consecutive items (and items separated by odd lags in general) which is partly in line with our earlier observations [3].

To better understand these off-diagonal patterns, we performed similar analyses as in our previous work [3] and examined whether off-diagonal patterns from overlapping representations might contribute to the patterns observed in the sequence matrix. For example, our prior research has shown that off-diagonal patterns in the random key matrix explained off-diagonal patterns from the motor sequence matrix in the motor cortical regions [3]. We therefore repeated the lag analyses described above for the across sequence matrix on the RD POS and RD ITEM matrices**.** For the **RD POS matrix**, the analysis revealed a main effect of lag in M1, PMC and PHC but not in the HC and PER (see S5A Fig RD POS for detailed statistics). Planned pairwise comparisons in M1 and PMC showed a similar pattern of results as in the SQ ACROSS, i.e., greater similarity values in lag 0 as compared to all other lags and greater dissimilarity values in lag 7 as compared to most other lags. As off-diagonal patterns were observed in both across sequence and random position matrices, we tested whether the lag patterns observed in the RD POS matrix was predictive of the lag patterns in the SQ ACROSS matrix. To do so, for each participant and ROI, we correlated the across sequence and random pos matrices using only off-diagonal cells (lags 1-7 averaged across upper and lower triangles of the respective matrices) and tested for each ROI whether the average correlation was significantly higher than zero (two-sided one sample t-test, corrected for the number of tests, N = 5 ROIs). The results are presented in S5B Fig and indicate that, similar to the results presented on the diagonal cells in the main text, SQ ACROSS off-diagonal data were significantly explained by the RD POS off-diagonal data in M1, PMC and PHC (M1: t_(29)_=5.82, *p_corr_*<.005; PMC: t_(29)_=6.4, *p_corr_*<.005; PHC: t_(29)_=2.79, *p_corr_*<.05) but not in PER (t_(29)_=1.4, *p_corr_*=.86) or HC (t_(29)_=.40, *p_corr_*=1). Altogether, these results indicate that the off-diagonal pattern observed across memory domains could be attributed to position coding in M1, PMC and PHC but not in the HC and PER.

Next, we tested for lag effects in the **RD ITEM matrix** and results did not show any lag-dependent effects in the random item matrix for any of the ROIs (see S5A Fig RD ITEM for detailed statistics). Correlation analyses show that the ACROSS SEQ off-diagonal data could not be explained by the RD ITEM off-diagonal data in any of the ROIs (M1: t_(29)_=1.19, *p_corr_*=1; PMC: t_(29)_=1.07, *p_corr_*=1; PHC: t_(29)_=-.01, *p_corr_*=1; PER: t_(29)_=-.02, *p_corr_*=1; HC; t_(29)_=-.24, *p_corr_*=1, see S5B Fig).

In sum, the results of the off-diagonal pattern analyses closely mirrored the results presented in the main text on the diagonal as they show that off-diagonal patterns across memory domains could be attributed to position coding in M1, PMC and PHC. Interestingly, off-diagonal patterns observed in the HC and PER could not be explained by other representations (i.e., position or item). In other words, the fluctuation in similarity values across lags in these regions was specifically related to the distance between items in the sequence (as opposed to simple position coding in the other ROIs). This therefore suggests that off-diagonal patterns in the HC and PER might reflect information about temporal order in a sequence across memory domains.

1. Surrogate analyses

We computed surrogate control matrices to provide an estimation of random / baseline data (see S6 Fig). For each individual, surrogate neural similarity matrices were created by randomly shuffling the labels of the fingers within the SQ MOT and RD KEY matrices, objects within the SQ OBJ and RD OBJ matrices, items within the SQ ACROSS and RD ITEM matrices and positions within the RD POS matrix (1000 permutations within each matrix). In a first step, we replicated the diagonal vs. off diagonal statistical analyses presented in the main text to assess a baseline effect on surrogate matrices (averaged across the 1000 permutations). Results show that there were no significant differences in mean pattern similarity between diagonal and off-diagonal cells of the surrogate matrices for any of the conditions in any of the ROIs (all ps_corr_>0.05, see S6 Fig for a depiction of the surrogate matrices and S7 Fig for diagonal vs. off-diagonal comparisons). These results indicate that the diagonal vs. off-diagonal patterns were not observed on baseline, random data. Next, we tested whether the observed pattern similarity effects reported in the current study exceeded what would be expected based on random noise (modelled in the surrogate matrices). To do so, matrices obtained in the main analyses were recalculated for each individual by subtracting their corresponding surrogate matrix (obtained by averaging over all 1000 permutations). All the analyses presented in the main text were repeated on these new matrices and the results are presented below. The results of these analyses showed identical effects as the original results presented in the main text. These analyses therefore indicate that the results reported in the main text were above chance / noise / baseline levels.

**Motor memory domain**

Results derived from the 8x8 ***SQ MOT*** matrix revealed significantly higher mean similarity along the diagonal (i.e., same finger + position) as compared to the off-diagonal (i.e., different finger + position) in all motor ROIs (paired sample t-test: M1, t(29)=9.7, d=1.8, p_corr_<0.005; PMC, t(29)=8.2, d=1.5, p_corr_<0.005; HC, t(29)=2.5, d=0.5, p_corr_=0.05). These results indicate that all motor ROIs carry information about fingers in their learned temporal position in the sequence. Results derived from the 8x8 ***RD KEY*** matrix revealed significantly higher mean similarity along the diagonal (i.e., same finger/key) as compared to the off-diagonal (i.e., different finger/key) in M1 and PMC (paired sample t-test: M1, t(29)=9.9, d=1.8, p_corr_<0.005; PMC, t(29)=7.2, d=1.3, p_corr_<0.005) but not in the HC (t(29)=-0.2, d=-0.04, p_corr_=4.2). To assess position coding irrespective of the finger, we computed similarity in activation patterns between individual positions in a sequence across repetitions of the random series. The 8x8 ***RD POS*** matrix showed significantly higher mean similarity along the diagonal (i.e., same position) as compared to the off-diagonal (i.e., different position) in M1 and PMC (paired sample t-test: M1, t(29)=5.7, d=1.05, p_corr_<0.005; PMC, t(29)=6.1, d=1.1, p_corr_<0.005) but not in the HC (t(29)=1.5, d=0.26, p_corr_=0.4). Altogether, these results indicate that M1 and PMC and carry information about finger and during random practice.

**Declarative memory domain**

Results derived from the 8x8 ***SQ OBJ*** matrix revealed significantly higher mean similarity along the diagonal (i.e., same object + position) as compared to the off-diagonal (i.e., different object + position) in all object ROIs (paired sample t-test: PHC, t(29)=5.6, d=1.02, p_corr_<0.005; PER, t(29)=2.9, d=0.54, p_corr_=0.02; HC, t(29)=3.7, d=0.68, p_corr_<0.005). These results indicate that all object ROIs carry information about objects in their learned temporal position in a sequence. Results derived from the 8x8 ***RD OBJ*** matrix did not reveal any significant difference in mean similarity along the diagonal (i.e., same object) as compared to the off diagonal (i.e., different object) in any of the object ROIs (PHC, t(29)=1.05, d=0.2, p_corr_=0.8; PER, t(29)=0.54, d=0.1, p_corr_=1.5; HC, t(29)=1.5, d=0.3, p_corr_=0.39). The 8x8 ***RD POS*** matrix revealed significantly higher mean similarity along the diagonal (i.e., same position) as compared to the off-diagonal (i.e., different position) for the PHC (t(29)=2.9, d=0.5, p_corr_=0.02) but not for the PER or the HC (PER, t(29)=0.5, d=0.1, p_corr_=1.5; HPC, t(29)=1.5, d=0.3, p_corr_=0.4). These results suggest that PHC carries position information.

**Domain-general effects (across memory domains)**

Results derived from the 8x8 ***SQ ACROSS*** matrix revealed significantly higher mean similarity along the diagonal (i.e., object/key + same position) as compared to the off-diagonal (i.e., object/key + different position) in all ROIs (paired sample t-test: M1, t(29)=7.2, d=1.3, p*_corr_*<0.005; PMC, t(29)=7.9, d=1.4, p*_corr_*<0.005; HC, t(29)=5.6, d=1.02, p*_corr_*<0.005; PHC, t(29)=6.8, d=1.2, p*_corr_*<0.005; PER, t(29)=6.3, d=1.1, p*_corr_*<0.005). These results indicate that all ROIs carry information about items (irrespective of their domain) in their learned position in the sequence. Results derived from the ***RD ITEM*** 8x8 matrix did not reveal any significant differences in mean similarity along the diagonal (i.e., object and key in the random condition but that were presented in the same temporal position in sequence condition) as compared to the off diagonal (i.e., objects and keys with different temporal positions in the sequence) in any of the ROIs (M1, t(29)=1.04, d=0.2, p*_corr_*=0.8; PMC, t(29)=0.5, d=0.1, p*_corr_*=1.5; HC, t(29)=1.6, d=0.3, p*_corr_*=0.3; PHC, t(29)=1.9, d=0.4, p*_corr_*=0.2; PER, t(29)=1.9, d=0.4, p*_corr_*=0.2). As a reminder, position coding results described above (8x8 ***RD POS***) indicate that M1, PMC and PHC carry information about position in random patterns.

1. Cross-validation analyses

To assess the reliability of Pearson correlation measures we used an approach recommended by [4] that assesses reliability using split-half reliability estimates. Specifically, we divided our data into two independent splits of odd and even runs and recomputed our similarity matrices within each half split. We next computed the similarity (using person correlation) between the matrices derived from each half split to assess reliability (higher correlations = higher reliability/reproducibility) for each condition (7 different matrices, i.e., motor sequence, object sequence, across sequence, random position, random key, random object and random item). Results showed that the RSA split-half reliability measures are in the range of what was previously observed for such representational similarity analyses [4]. As expected, reliability was greater across all ROIs for the specific matrices testing for the conditions coded by the specific ROIs. In other words, if a particular brain region represented a particular type of information, the reliability of the measures within the matrix corresponding to this particular condition was greater than for matrices corresponding to conditions that were not represented by the brain region (see S8 Fig). Specifically, all ROIs showed a main effect of matrix (M1: F(6,162)=45.72, ɳ_p_^2^=.63, p<.001; PMC: F(6,162)=38.06, ɳ_p_^2^=.59, p<.001; PHC: F(6,162)=14.65, ɳ_p_^2^=.35, p<.001; HC: F(6,162)=12.67, ɳ_p_^2^=.32, p<.001; PER: F(6,162)=8.12, ɳ_p_^2^=.23, p<.001; see S8 Fig for pairwise comparisons between conditions). Specifically, M1 showed higher reliability for finger coding compared to all other matrices (all p_corr_<.05), for finger-position coding compared to all other matrices excluding the item-position matrix (all p_corr_<.01) and for object- and item-position coding compared to object, item and/or position coding (all p_corr_<.01). The PMC showed higher reliability for finger- and item-position coding than for finger, pos, object and item coding (all p_corr_<.05) and for object-position, finger and position coding than object and item coding (all p_corr_<.01). The PHC showed higher reliability for finger-position binding than object and item coding (all p_corr_<.05) and for item-position binding than finger, position, object and item coding (all p_corr_<.01). The HC shower higher reliability for item-position coding than all other matrices (all p_corr_<.05). Last, the PER showed higher reliability for item-position coding than for finger, position, object and item coding (all p_corr_<.05). Importantly, the results show overall moderate to good reliability for the matrices corresponding to the information coded in the specific brain regions described above.

1. Eye movement analyses

Gaze coordinates were extracted from each volume across all runs and participants using the DeepMReye toolbox [5]. DeepMReye uses a convolutional neural network trained to decode gaze location directly from the MR signal of the eyeballs. To generate gaze estimates for each fMRI volume, we applied a set of publicly available pre-trained weights, provided by the developers of the toolbox. These weights were originally trained on datasets that included simultaneous BOLD fMRI and eye-tracking data, enabling reliable inference of gaze direction without the need for in-scanner eye tracking. DeepMReye classifies ten gaze coordinates per functional volume. These gaze coordinates were used to conduct two control analyses to assess *(1) Mean distance travelled by the eyes (2) Patterns related to the visual processing of the central object.* The goal of these control analyses was to compare gaze patterns between the different task conditions to better characterize the nature of object sequence learning.

First, we computed the distance traveled by the eyes in the three conditions (motor, object and random) as well as during rest periods (when participants are instructed to fixate the center of the screen) in the 8 different runs. For each condition and run, distance measures were normalized by the total number of measurements for the specific condition and run. We tested whether the quantity of eye movements (mean distance) across the 8 runs differed between task (across the three different conditions) and rest periods, as well as among the three conditions of interest. Results of a 2 (task vs. rest) x 8 (run) repeated-measures ANOVA showed that, as expected, there was a significant difference in mean distance between task and rest, with greater distance travelled during task as compared to rest (task effect: F(1,27)=32, ɳ_p_^2^=.54, p<.001). This effect was stable across runs (run effect: F(7,189)=.76, ɳ_p_^2^=.03, p=.62; task by run effect: F(7,189)=1.83, ɳ_p_^2^=.06, p=.08; S9A Fig). Results of a 3 (condition: motor, object and random) x 8 (run) repeated-measures ANOVA on mean distance showed no main effect of condition or run and no condition by run interaction (condition by run effect: F(14,378)=1.24, ɳ_p_^2^=.04, p=.25; condition effect: F(2,54)=.61, ɳ_p_^2^=.02, p=.54; run effect: F(7,189)=1.42, ɳ_p_^2^=.05, p=0.2; S9B Fig) suggesting that the quantity of eye movements did not differ between task conditions. Taken together, these results suggest that eye movements were not a confounding factor between conditions in this paradigm.

Next, we compared heat maps of gaze coordinates between the 4 different task conditions mentioned above. Heat maps for each condition were built for each run with a resolution of 0.5° × 0.5° visual angle pixels. Each pixel was assigned a value corresponding to the number of measurements in which gaze position was recorded within this area. The resulting heatmaps were then normalized by the total number of measurements in each condition and each run and were then averaged across runs (see S10A Fig for separate condition heatmaps averaged across runs). A cluster-based permutation approach using 10,000 permutations and a p-value threshold of 0.05 was used to identify significant spatial clusters where gaze patterns differed significantly between pairs of conditions. The results of the cluster analysis are overlaid on the differential heat maps in S10B Fig. Results revealed that there was no significant difference in gaze patterns between the 2 sequence conditions (motor and object). This suggests that participants used similar visual search strategies for the two sequence conditions. Interestingly, participants spent less time fixating the center of the screen during the object condition as compared to the random condition. These findings suggest that the knowledge of the series of objects might have resulted in a decreased need to fixate the central cue to complete the task. Note that there was no difference in gaze patterns between the motor sequence and the random condition. Last, the comparison of gaze patterns between task conditions (collapsed across motor sequence, object sequence and random conditions) and rest periods revealed greater central fixation during rest compared to task (participants were instructed to fixate a central square during rest) and greater lateral eye movements during task compared to rest (presumably due the visual exploration of the object/key mapping).

Altogether, the results of the eye movement analyses suggest that eye movements were not a confounding factor between conditions and revealed two key findings that suggest that (i) participants used a similar visual search strategy for the two sequence conditions, and (ii) the knowledge of the series of objects resulted in a decreased need to fixate on the central cue to complete the task as compared to the random condition.

References

[1] Hsieh L-T, Gruber MJ, Jenkins LJ, Ranganath C. Hippocampal activity patterns carry information about objects in temporal context. Neuron 2014;81:1165–78.

[2] King BR, Dolfen N, Gann MA, Renard Z, Swinnen SP, Albouy G. Schema and motor-memory consolidation. Psychol Sci 2019;30:963–78.

[3] Dolfen N, Reverberi S, Op de Beeck H, King BR, Albouy G. The hippocampus represents information about movements in their temporal position in a learned motor sequence. J Neurosci 2024;44:e0584242024.

[4] Walther A, Nili H, Ejaz N, Alink A, Kriegeskorte N, Diedrichsen J. Reliability of dissimilarity measures for multi-voxel pattern analysis. Neuroimage 2016;137:188–200.

[5] Frey M, Nau M, Doeller CF. Magnetic resonance-based eye tracking using deep neural networks. Nat Neurosci 2021;24:1772–9.
